# Stress and Strain: Differentiating the Responses to High and Moderate Heat Loads and Subsequent Recovery in Grain-Fed Feedlot Steers—Plasma Biochemistry

**DOI:** 10.3390/ani16091379

**Published:** 2026-04-30

**Authors:** Gene Wijffels, Sally Stockwell, Suzie Briscoe, Yutao Li, Russell McCulloch, John B. Gaughan

**Affiliations:** 1 CSIRO Agriculture and Food, Queensland Bioscience Precinct, Brisbane, QLD 4067, Australia; 2 School of Agriculture and Food Sustainability, The University of Queensland, Gatton, QLD 4343, Australia; j.gaughan@uq.edu.au

**Keywords:** feedlot cattle, heat stress, metabolism, ischemic reperfusion injury, hypoperfusion

## Abstract

The extent of, and limits of, the metabolic flexibility of feedlot cattle to cope with heat loads of varying intensity and duration is a research gap. Two cohorts of 12 Black Angus steers were housed in climate-controlled rooms (CCR) and subjected to three thermal periods: PreChallenge (5 days), Challenge (7 days) and Recovery (5 days). PreChallenge and Recovery provided thermoneutral conditions. The Challenge simulated a strong heatwave. Finally, the steers were returned to outdoor pens for 20 days. The animals were bled on days 3, 5, 7, 8, 9, 10, 11, 12, 13, 15, 17, 24 and 38. A clinical plasma biochemistry panel was used to measure the concentrations of major metabolites and electrolytes. During Challenge, energy metabolites fell (except for β-hydroxybutyrate). Creatinine, urea and total bilirubin rose rapidly. In Recovery, the major liver enzymes were released into plasma, and total bilirubin remained high. Most analytes showed non-linear relationships with core temperatures during Challenge, suggesting threshold-dependent responses rather than gradual dose-dependent adjustments. The responses and relationships differed from those reported for moderate heat load challenge and recovery. We integrated the metabolic changes over the course of the experiment with previously reported metabolic hormone and physiological responses of these steers.

## Simple Summary

Rising summer temperatures, along with greater frequency, intensity and duration of moderate and severe heatwaves, are negatively impacting livestock welfare and productivity. We are investigating the immediate and post-heatwave effects (strain) of different levels of heat stress on grain-fed feedlot Black Angus steers. This study follows the changes in the major plasma metabolites and electrolytes during and after a high heat load challenge. The responses were highly dynamic and system-wide. With blood flows directed to the skin and away from the organs, metabolic rate is lowered to reduce heat production by the body. Recovery is not immediate and reveals signs of tissue damage that occurred during the Challenge. In our previous study of a moderate heat load challenge, the steers adjusted by reducing feed intake, increasing drinking and sweating. In recovery, physiologically and metabolically, they soon returned to normal. However, the high heat load challenge appears to push the steers into a new, less productive physiological state in recovery and sometimes after.

## 1. Introduction

The livestock production industries worldwide recognize the effects of global warming on their production systems and animal welfare [1,2]. There are a limited number of dependable changes observed in heat-stressed ruminants—raised respiration rates (RR) and core temperature, reduced dry matter intake (DMI) and weight gain, increased water intake and in the case of dairy cattle, decreased milk production. However, across the ruminant heat stress research literature, the reports of responses from any other number of lenses (metabolic, endocrine, immune/inflammatory) are highly variable and often contradictory. There are many, many good reasons for this circumstance. Studies range from seasonal heat stress on pasture where animals are followed over multiple heat stress events, to climate-controlled room experiments investigating a single thermal challenge. In both situations, the nature of the thermal challenges differs widely in intensity and duration. Sampling and observation schedules may range from simple before and after thermal challenge time points to more intensive regimes. The animal cohorts can be vastly different: beef vs. dairy, age, stage of lactation in dairy cattle, and different breeds. The high-producing dairy cow is particularly vulnerable to heat stress. Its responses to heat stress and management continue to be a subject of much research [3,4]. By comparison, responses and capacity of beef cattle to cope with heat stress are understudied [5]. Moreover, to ensure rapid growth, feedlot steers are fed high-energy diets, which lead to increased core temperatures relative to pastured cattle, thus increasing the vulnerability of feedlot animals to heat stress [6]. On the other hand, the resilience of the Brahman breed (*Bos indicus*) to thermal challenge is remarkable [7].

To disentangle and somewhat quantify the responses (strain) to varying heat load (stress), we conducted two experiments in CCR on grain-fed Black Angus steers, the mainstay of the Australian beef industry [8]. The thermal regime in each experiment consisted of a thermoneutral period (PreChallenge), a heat load period (Challenge), a recovery period with a return to thermoneutral conditions (Recovery) and finally a follow-up in outdoor feedlot pens (PENs). The first experiment, the moderate heat load (MHL) experiment, delivered a consistent thermal challenge of 7 days of diurnal cycling between 28 and 35 °C (temperature-humidity index (THI): 73.6–82.5). The thermally challenged (TC) steers were contrasted with feed-restricted thermoneutral (FRTN) animals run concurrently in the same facility [9]. The findings of the MHL experiment have been described [9,10,11,12]. In brief, we found the steers ably coped with the conditions. The physiological changes occurred, and at the endocrine and metabolic levels, many parameters changed in a linear fashion with core temperatures or DMI. We concluded that the steers coped with MHL by using homeorhetic mechanisms to adjust to strain and to recover. They returned to similar feed intake, growth rates and live weights as their FRTN counterparts. Bauman and Currie (1980) [13] described homeorhesis as “the orchestrated or coordinated changes in metabolism of body tissues necessary to support a physiological state”. It allows an animal to alter their physiological state to adjust to a new environment or stressor, with the degradation of important functions such as growth and reproduction [14]. As such, the animal maintains functional integrity [15]. When the stressor diminishes, the adjustments are reversible.

The physiological responses to the HHL challenge, and critically those in Recovery, differed from those of the MHL experiment. We argued that the steers entered an allostatic state in Recovery and remained so to some extent in the final outdoor period [16]. Subsequent analyses of their endocrine responses tended to support this premise [17]. Allostasis is a means of adaptation to ongoing and/or severe stress that may give rise to new physiological set points and learned responses [15,18,19]. If the load is too high, the pathology may occur [18,20].

**Figure 1:**
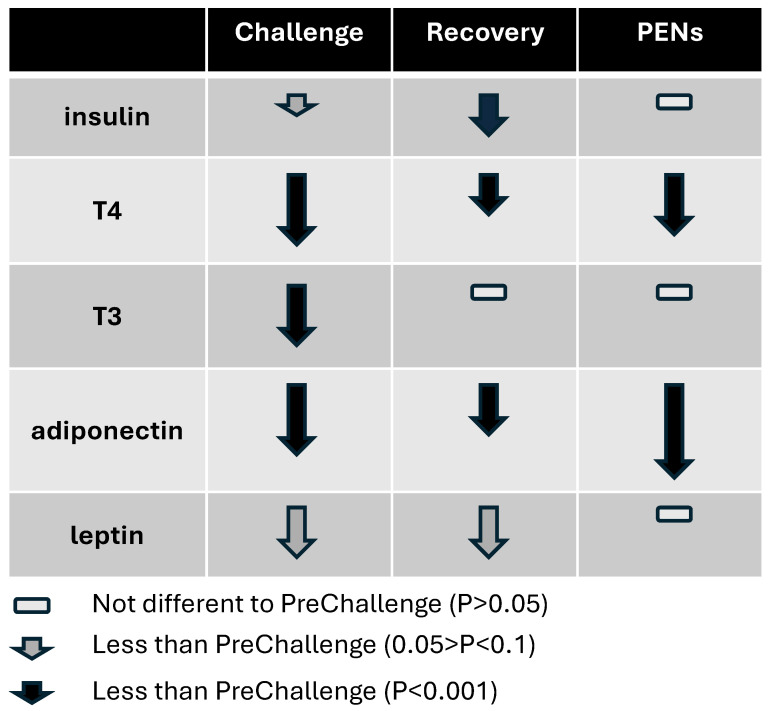
A schematic showing the responses of the metabolic hormones during the high heat load (HHL) experiment [17]. No difference or a reduction in mean hormone concentration for the Challenge, Recovery and PENs periods relative to the PreChallenge is displayed. The length of the arrow is indicative of the magnitude of the reduction in mean concentration. The shading of the arrow indicates the level of significant difference with the PreChallenge mean. For example, the Challenge mean insulin concentration was 5% less than the PreChallenge concentration (*p* = 0.0897).

The endocrine milieu during the HHL Challenge was dominated by substantially reduced T3 and T4, and adiponectin concentrations, and slight depressions in insulin and leptin concentrations (ref. [17], see Figure 1 and Supplementary Figure S1). The lower levels of thyroid hormones restrain metabolic activity in most organs. The impacts of the altered levels of the hormones on hepatic energy metabolism were difficult to discern due to the often-contradictory influences of these hormones in the liver. In skeletal muscle, the hormone milieu would have depressed energy metabolism and protein synthesis. In adipose, there would be a similar scenario alongside reduced lipolysis and lipogenesis.

The objectives of the study were firstly to identify the metabolic effects of HHL on feedlot cattle by way of a clinical biochemistry panel, and secondly, to uncover the dynamics of the analyte concentrations in relation to each other and to core temperature. Thirdly, we looked for associations with the behaviors of the metabolic hormones investigated previously for this experiment [17]. In doing so, we hoped to gain an understanding of which major organs were affected by HHL and for how long (the strain). For example, in Recovery, how quickly do the organs respond to falling core temperatures and return to normal function, or is there an adjustment, or a resetting of function? Furthermore, we went on to compare the responses of HHL with those of MHL.

## 2. Materials and Methods

### 2.1. Animal Experiment

Receipt and preparation of the Black Angus steers are described in detail in [16]. On arrival, the steers were 16–18 months of age. The experiment was conducted on site at the Queensland Animal Science Precinct (Gatton, Queensland, Australia) over the summer through to autumn of 2017 (January–May). The animal ethics protocol was specifically developed for this experiment and approved by the University of Queensland animal ethics committee (certificate number SAFS/460/16).

The steers were subjected to two sequential stages prior to the experiment. During the first stage of induction and backgrounding, the cattle were maintained on pasture, underwent an industry-standard vaccination regime, and were treated for parasites over a period of 4 weeks. At the end of this stage, 24 steers were selected to make up two cohorts of 12 animals. The cohorts (cohorts 1 and 2) were balanced by body weight, age and temperament. The second stage was a 60-day feedlot phase. Cohort 1 animals were moved to outdoor feedlot pens and administered a hormonal growth promotant implant and a radio-transmitting rumen temperature bolus [16]. Cohort 2 animals remained on pasture for a further 3 weeks. At feedlot entry, the non-fasted mean body weight (±SD) of the cohort 1 animals was 490.8 ± 32.3 kg/head, and that of the cohort 2 animals was 495.4 ± 35.9 kg/head. In the feedlot stage, the steers were gradually introduced to a grain-based feedlot diet and feed routine. At the end of this stage, the steers were adjusted to a feedlot finisher diet. The diet was composed of 87% grain, 9% cotton seed and 4% Lucerne hay. On a nutritional basis, dry matter was 90%, with 15% protein, 9% crude fiber, 5% fat and 35% starch (for more details, refer to [16]). This composition gave metabolizable energy of 13 MJ/kg, and Net Energy for Gain (Ne_g_) of 3.0 MJ/kg. At the end of the feedlot stage, the cohorts entered the CCR three weeks apart.

The housing capacity of the CCR was 12 animals. The configuration consisted of 4 rooms, each with 3 pens. Each steer was allocated a pen with a feed trough and an automated refilling water dish. The experimental period in the CCR was 17 days. On day 0, the steers entered the CCR. Day 1 was the start of the experiment. The steers exited the CCR on day 18. The following three days were used to clean and prepare the CCR for the next cohort. This schedule necessitated the 3-week separation of the entry to CCR by the two cohorts.

**Figure 2:**
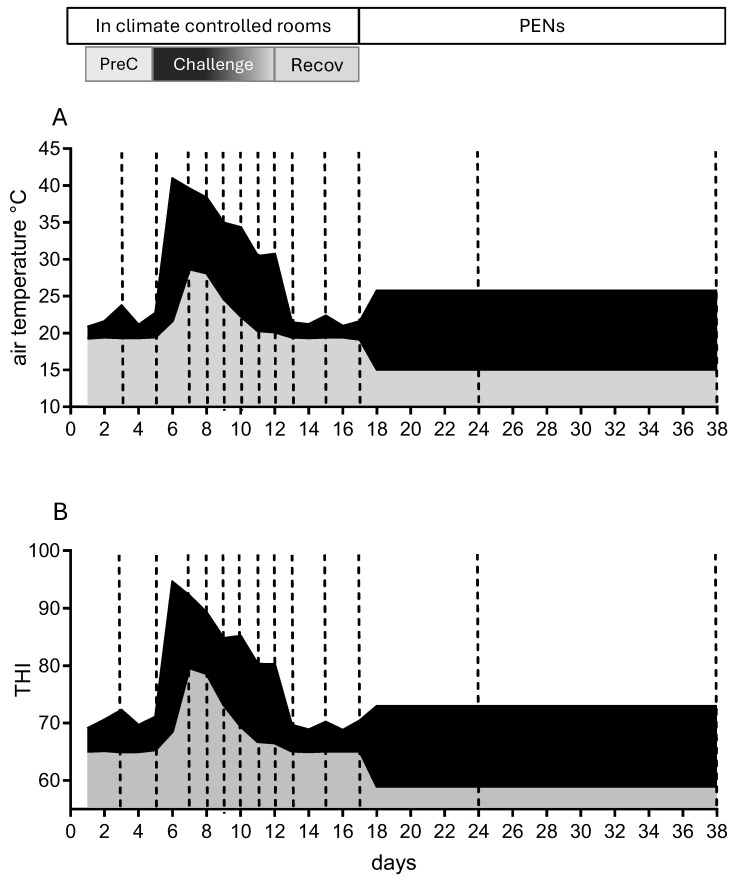
Black Angus steers were subjected to three consecutive periods of differing climatic conditions in climate-controlled rooms. PreChallenge (PreC, days 1 to 5) delivered thermoneutral conditions. Challenge (days 6 to 12) imposed a high heat load thermal challenge, and Recovery (Recov, days 13 to 17) returned the cattle to thermoneutral conditions. In PENs (days 24 to 38), the steers experienced benign ambient weather, shown as the average conditions in the schematic. The plots show the daily means of maximum (black filled profile) and minimum (gray filled profile) for: (**A**) Ambient temperature (TA, °C). (**B**) Temperature humidity index (THI). Blood sampling was conducted on days 3, 5, 7, 8, 9, 10, 11, 12, 13, 15, 17, 24 and 38 as indicated by the dashed vertical lines.

The two cohorts (*n* = 12) entered the CCR three weeks apart in March 2017 with a mean live weight of 603.4 ± 38.6 kg and a mean daily DMI of 13.3 ± 1.6 kg/head/day. The steers were housed in the CCR for 17 days and were subjected to three periods of differing climatic conditions (Figure 2). PreChallenge occurred over days 1–5 and provided near constant thermoneutral conditions with the mean (±SEM) daily TA at 20.7 ± 0.27 °C and mean daily THI at 67.8 ± 0.29.

During the Challenge period, the thermal load was delivered with diurnal cycling over days 6–12. The steers were abruptly introduced and maintained in high heat load conditions over days 6–8 (Figure 2). For days 6–8, the daily TA maximum range was 38.3–40.8 °C (daily THI maximum range of 89.1–94.5; Figure 2B) and the day 7 and 8 overnight TA minima were 28.7 and 28.1 °C, respectively (THI minima of 79.6 and 78.6). The conditions were eased on days 9 and 10 with daily TA maxima of 34.9 and 34.3 °C and daily TA minima of 24.7 and 22.3 °C, respectively (THI maxima: 85.1 and 84.8; THI minima: 73.1 and 69.4). These conditions for the first five days of Challenge fall into the Emergency THI category [21]. The last two days of the Challenge, days 11 and 12, conditions were further eased but still imposed the Danger category (THI 79–83). The daily TA maxima were 30.7 and 30.4 °C, and daily TA minima of 20.3 and 20.1 °C, respectively (THI maxima: 80.2 on both days; and THI minima: 66.8 and 66.5). Daily maximum and minimum percent relative humidity (%RH) are given in Supplementary Table S1.

Given the severity of the thermal challenge, procedures were put in place to ensure animal welfare. During the Challenge, each animal was monitored on site for 24 h of each day by real-time video and rumen temperature data from the wireless rumen bolus (with 10 min delay). The near-real-time rumen temperature data enable detection of dysregulated rumen temperature (RumT ≥ 41.5 °C; sudden escalation of temperature) and absence of water consumption. The video information allowed assessment of panting, body posture and neurological changes. Furthermore, hourly in-person inspection and records of each animal were conducted by experienced personnel to assess panting score, respiration rate (RR), water usage, body posture and flight score. Animals with RR above 120 bpm were reassessed every 30 min. The criteria for an adverse reaction to the thermal conditions were a combination of one to two hours of RumT ≥ 41.5 °C, panting score > 3.5, depressed posture and minimal response to stimuli. Under these criteria, two steers from each cohort were removed from the CCR and withdrawn from the experiment. Distressed animals were removed from the CCR, cooled with air fans under full shade until RumT approached normal.

In the Recovery period, days 13–17, thermoneutral conditions were resumed with mean (±SEM) daily TA at 20.4 ± 0.12 °C and mean daily THI at 67.3 ± 0.18. On exit from the CCR, the steers were returned to outdoor feedlot pens for a 20-day finishing phase (till day 38, Figure 2). The purpose of this phase was to allow the animals to recover, gain weight and be ready for sale. This is best achieved with minimal interference with the animals. In the seasonal cooling conditions, the mean maximum daily TA were within mid-20 °C, and daily minima ranged between 10 and 20 °C.

### 2.2. Clinical Biochemistry

To obtain plasma biochemistry data, the animals were bled by jugular venipuncture into 4 mL Vacutainer blood collection tubes containing either lithium heparin or potassium EDTA. The tubes were supplied by IDEXX Laboratories Pty Ltd. (Brisbane, Australia). The steers were bled at 7:30 AM on days 3, 5, 7, 8, 9, 10, 11, 12, 13, 15, 17, 24 and 38 (Figure 2). The blood sampling was conducted after completion of the 6:00 and 7:00 AM physiological observations (RR, panting score, overnight water usage, and feed refusals) and before the heat load from the programmed rise in morning air temperature (starting at 7:00 AM) started to impact the cattle. Immediately after venipuncture, each animal was returned to its individual pen and their feed allocation was provided.

The blood samplings on day 3 and day 5 were designed to determine a baseline concentration for the analytes in the PreChallenge thermoneutral conditions. As described above, the thermal challenge regime delivered a sudden increase in heat load on day 6. The steers were not bled on that day to avoid any further stress. The intent of the bleeds on days 7–12 during the Challenge was to capture the changes in the analytes as the steers responded to the simulated heatwave conditions. The blood samplings on days 7 and 8 reported analyte responses to daily maximum THI conditions of 89–94; the samplings on days 9 and 10 reported the analyte responses to daily maximum THI of 85; and the samplings on days 11 and 12 reported the responses to daily maximum THI of 80. There were three bleeds (days 13, 15 and 17) during the 5-day Recovery period in constant thermoneutral conditions (THI of 67). The rationale for fewer bleeds in this period was, in part, to allow the animals to recover, and with the expectation that most analytes would return to PreChallenge levels in the consistent benign conditions. The steers were bled twice during the PEN period when brought in for weighing. At the time of the experiment, the PEN period was not a focus of the study.

The filled 4 mL blood tubes were placed in a tube rack at ambient temperature until venipuncture of all the animals of the cohort was completed (approximately 30 min). The rack of blood tubes was placed in a lidded foam polystyrene box with prechilled (4 °C) ice packs, which in turn was placed in a refrigerator (4 °C). Two hours after blood collection, the enclosed foam box of blood tubes and ice packs was put in an air-conditioned vehicle (21 °C) for delivery to IDEXX Laboratories Pty Ltd. (Brisbane, Australia). On average, the blood tubes were received by the service laboratory for clinical chemistry analyses three hours after blood collection. Transporting of blood for clinical chemistry analysis at 4 °C within 24 h of collection is recommended [22]. Twenty-one analytes were measured for each bleed from each animal: concentrations of albumin, bicarbonate, β-hydroxybutyrate (BOHB), calcium, chloride, cholesterol, creatinine, glucose, magnesium, phosphate, potassium, sodium, total bilirubin, total protein, urea, and the activities of alkaline phosphatase (ALP), aspartate aminotransferase (AST), creatine kinase (CK), γ-glutamyl transferase (GGT) and glutamate dehydrogenase (GLDH). Globulin concentration was calculated as [total protein] − [albumin].

### 2.3. Statistical Analysis

#### 2.3.1. Generalized Linear Model

The data from the twenty animals that completed the trial were used in the statistical analyses. The approach followed that described in [17]. A Generalized Linear Model (GLM) of Analysis of Variance was performed on all the analytes using the SAS statistical software (version 9.4, TS1M1. SAS Institute Inc., Cary, NC, USA). The data from each analyte were assessed for the significance of three main effects and their interactions on the analyte. These include: cohort difference (2 levels), room variations (4 levels), period variations (4 levels), and two interaction terms: a nested effect of a room within a cohort (room(cohort)), and a nested effect of a period within a cohort (period(cohort)). A full GLM for an analyte can be presented as follows:

Y_ijkl_ = µ + C_j_ +R_k_ +P_l_ + R_k_(Cj) + P_l_(C_j_) + e_ijkl_

where Y_ijkl_ is the observed trait value of the ith animal from the jth cohort, kth room and lth period, µ is the population mean, C_j_ represents the jth cohort (j = 1 or 2), R_k_ refers kth room (k = 1, 2, 3 or 4), and P_l_ indicates lth period (l = 1, 2, 3, or 4). R_k_(C_j_) and P_l_(C_j_) are the nested interaction terms for R_k_ or P_l_ within the ith cohort. e is a random error term.

The LSMEANS statement in SAS GLM was applied: (a) to compute the least-square means and the least-square effects for all fixed effects in the model, and (b) to conduct multiple pair-wise comparisons of individual levels of the main effects and interactions to derive corresponding *p*-values. All non-significant main effects and interactions were removed from the model. The predicted (fitted) values were then derived from the final models for further analysis. The residues were examined for normality using the Shapiro–Wilk test in the R Studio 2024.04.2 Build 764 program. They were also compared with the predicted values from the ANOVA to ensure there was no clear relationship between them.

#### 2.3.2. Regression Analyses

Regression analyses were conducted in Prism 10.0 (GraphPad Software, San Diego, CA, USA). Linear regression was assessed on the Pearson correlation coefficient (r) and level of significance (*p*). *p*-values less than 0.05 were considered significant, and *p*-values in the range of 0.05 to 0.1 were assessed as trending towards significance. Non-linear regression (quadratic or exponential) was assessed with the coefficient of determination (R^2^). When comparing linear and non-linear models of regression, the R^2^ values, values of the standard deviation of the residuals (Sy.x) and Corrected Akaike Information Criterion (AICc) were compared. The model with the greatest R^2^ value, and the least AICc and Sy.x values was selected. To ensure all selected models were of good fit, the *p*-values from Run Tests were assessed, and the difference between the adjusted R^2^ value and the standard R^2^ value was examined.

## 3. Results

The analyses below describe the metabolite concentrations and enzyme activity trajectories, the comparison of period means, and the relationships with core temperatures (RumT and RecT). The analysis extends to correlations between the metabolite concentrations (Supplementary Tables S2 and S3, and Supplementary Figures S4, S8, S11, S12, S16 and S17) and with the metabolic hormone concentrations previously reported (ref. [17], Supplementary Tables S4 and S5, and Supplementary Figures S3, S6, S9, S13 and S16). Cross-correlations were conducted to develop an understanding of the concurrent changes in the analytes/enzymes in the HHL experiment.

### 3.1. Energy Metabolites

**Figure 3:**
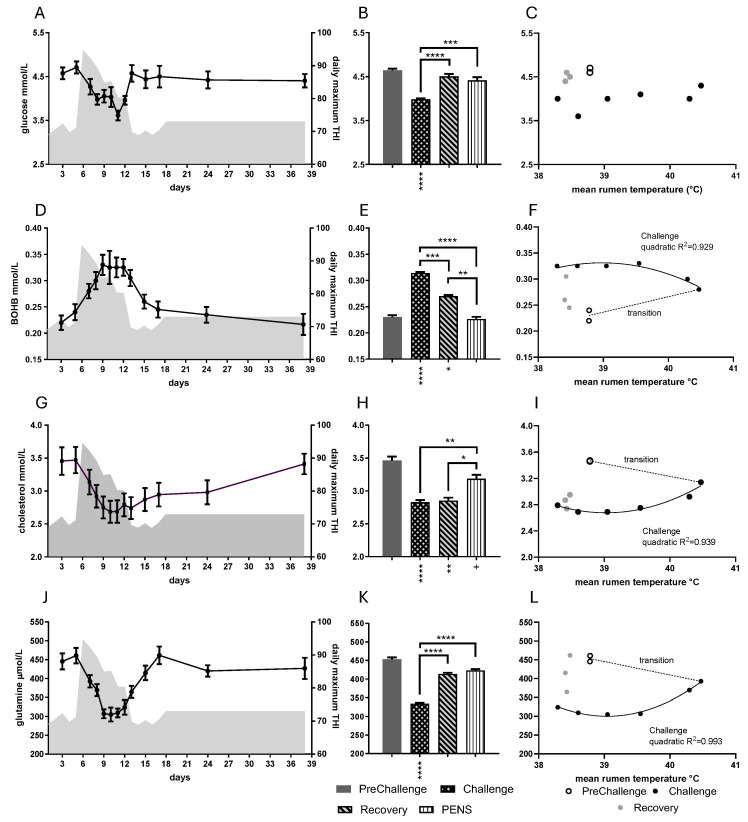
Behavior of energy metabolite concentrations: glucose, BOHB, cholesterol and glutamine. Panels (**A**,**D**,**G**,**J**): Trajectories of the daily mean (±SE) of each metabolite over the 38-day trial underlaid with the daily maximum THI profile (shaded area). Note that the line connecting the data points is interpolated. Panels (**B**,**E**,**H**,**K**): Histograms of the period means (±SE) of each metabolite during PreChallenge, Challenge, Recovery and PENs. Significant differences with the PreChallenge mean are indicated by asterisks below the *x*-axis. Significant differences amongst the later periods are denoted by asterisks above the brackets. +, *p* < 0.10; *, *p* < 0.05; **, *p* < 0.01; ***, *p* < 0.001; **** *p* < 0.0001. Panels (**C**,**F**,**I**,**L**): Plots of the relationships of the daily mean of each metabolite with rumen temperature. Where applicable, the transition from the PreChallenge mean to the first day of Challenge is indicated (dotted line). The coefficient of determination (R^2^) is displayed where a quadratic relationship was found.

There were strong effects of period on the concentrations of the energy metabolites (*p* < 0.0001 in all cases). Each presented a unique trajectory over the 38-day trial (Figure 3). Overall, there was a strong correlation between metabolite concentrations. In Challenge, daily mean glucose concentration appeared to fall in a two-step manner; the lowest concentration, 3.16 mM, was attained on day 11 (Figure 3A). In the later periods, glucose concentration recovered to be close to PreChallenge concentrations. The Challenge period mean was the least of all the period means (Figure 3B, Table 1). There were no relationships evident for daily mean glucose with the core temperatures (Figure 3C; Supplementary Figure S2A) nor with any of the metabolic hormones, including insulin.

Daily mean β-hydroxybutyrate (BOHB) concentration rose during Challenge, achieving a plateau over days 9–12. It returned to near PreChallenge levels at the end of Recovery (Figure 3D). The mean BOHB concentrations during Challenge and Recovery were greater than those of PreChallenge (Figure 3E, Table 1). There were strong quadratic relationships (a shallow inverted parabola) with core temperatures during Challenge (Figure 3F; Supplementary Figure S2B). RumT was at maximum on day 7 (40.47 °C); however, the mean BOHB concentration continued to rise till day 9 (RumT: 39.55 °C). It remained at this level even as RumT fell (day 12). BOBH was the only energy metabolite to show an association with any of the metabolic hormones (Supplementary Tables S4 and S5). There were overall negative linear correlations with T3 (Supplementary Figure S3A) and T3 log10 concentration (Supplementary Figure S3B).

Daily mean cholesterol concentration decreased steadily during Challenge, coming to a minimum on day 10 (at 2.69 mM). Thereafter, it rose very gradually, eventually returning to PreChallenge concentrations on day 38 (Figure 3G). The mean concentrations during Challenge, Recovery and PENs were less than the PreChallenge mean (Figure 3H, Table 1). Daily mean cholesterol also exhibited strong quadratic relationships with core temperatures during the Challenge (Figure 3I; Supplementary Figure S2C). Cholesterol concentration was steady over days 9–12 as RumT fell (39.55 to 38.60 °C), and in Recovery, it remained low despite low RumT (Figure 3I).

Daily mean glutamine concentration fell rapidly early in Challenge, before returning to PreChallenge concentrations (Figure 3J). At the level of the periods, the Challenge mean was less than the PreChallenge mean (Figure 3K, Table 1). Daily mean glutamine concentrations exhibited strong quadratic relationships with core temperatures during Challenge (Figure 3L; Supplementary Figure S2D). The most change occurred over days 7–9, whereupon it stabilized over days 9–11 (Figure 3L). The consistently low RumT in Recovery (38.41– 38.47 °C) saw the glutamine concentration increase in 40–50 mM increments through days 13, 15 and 17.

There were high levels of cross-correlation between the mean daily concentrations of the energy metabolites (Supplementary Tables S2 and S3, Supplementary Figure S4). Over the 38 days of the trial, and during the CCR periods, glucose concentration was strongly negatively correlated with BOHB but strongly positively correlated with glutamine concentration (Supplementary Figure S4A,B). Neither relationship was maintained in Challenge. BOHB concentration was also negatively correlated with cholesterol and glutamine concentrations (Supplementary Figure S4C,D). Finally, cholesterol concentration was strongly positively correlated with glutamine concentration overall and during Challenge. (Supplementary Figure S4E.)

### 3.2. Creatinine and Urea

**Figure 4:**
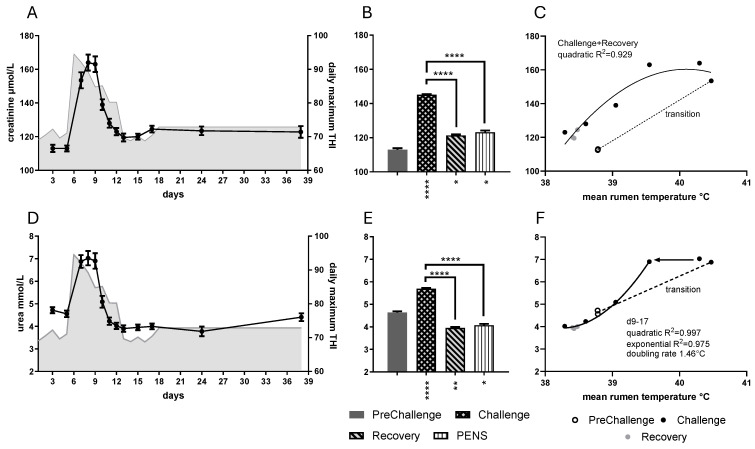
Behavior of creatinine and urea concentrations. Panels (**A**,**D**): Trajectories of the daily mean (±SE) of the metabolites over the 38-day trial underlaid with the daily maximum THI profile (shaded area). Note that the line connecting the data points is interpolated. Panels (**B**,**E**): Histograms of the period means (±SE) of the metabolites during PreChallenge, Challenge, Recovery and PENs. Significant differences in the period mean with the PreChallenge mean are indicated by asterisks below the *x*-axis. Significant differences amongst the later periods are denoted by asterisks above the appropriate brackets. *, *p* < 0.05; **, *p* < 0.01; ****, *p* < 0.0001. Panels (**C**,**F**): Plots of the relationships of the daily mean of each metabolite with rumen temperature. Where applicable, the transition from the PreChallenge mean to the first day of Challenge is indicated (dotted line). The coefficient of determination (R^2^) is displayed where quadratic or exponential relationships were found.

Creatinine and urea concentrations rose rapidly with the sudden onset of heat load and subsided as quickly when conditions cooled. Not surprisingly, there were strong effects of period on creatinine and urea concentrations (*p* < 0.0001 in both cases; Figure 4). Creatinine rose rapidly to peak on days 8 and 9, subsequently falling to stay within the narrow range from day 12 to day 38 (Figure 4A). The mean creatinine concentrations for Challenge, Recovery and PENs were all greater than the PreChallenge mean (Figure 4B, Table 1). Creatinine concentration displayed strong quadratic relationships with core temperatures over Challenge and Recovery (Figure 4C; Supplementary Figure S5A). Creatinine concentration fell as RumT arrived at 39.04 °C (day 10).

Daily mean urea concentration also rose rapidly and achieved a plateau at 6.88–7.03 mM over days 7–9 (Figure 4D). The mean urea concentration during Challenge was greater than the PreChallenge mean, whereas the Recovery and PENs were less (Figure 4E, Table 1). Urea concentration showed a strong linear relationship with RumT (Supplementary Figure S5B). Inspection of the plot suggested a biphasic response due to the plateau over Challenge days 7–9. After day 9, and through till day 17, there was rapid decay described by a quadratic model (Figure 4F). An exponential function calculated the doubling rate at 1.46 °C RumT. Thus, as mean daily RumT fell below 39.55 °C (days 9–10), the urea concentration fell by half for every 1.46 °C RumT till it stabilized below the PreChallenge mean concentration. Similar behavior was observed with RecT (Supplementary Figure S5C).

Daily mean urea and creatinine concentrations were strongly and positively correlated (Supplementary Figure S5D). Creatinine concentration had a strong negative linear correlation with T4 concentration (Supplementary Figure S6A), but a quadratic relationship was also tenable (Supplementary Figure S6B). Urea and creatinine concentrations returned linear relationships with bicarbonate, chloride, sodium and calcium concentrations (Supplementary Tables S2 and S3, see more below). There were no associations with metabolic hormone concentrations.

### 3.3. Liver Function

**Figure 5:**
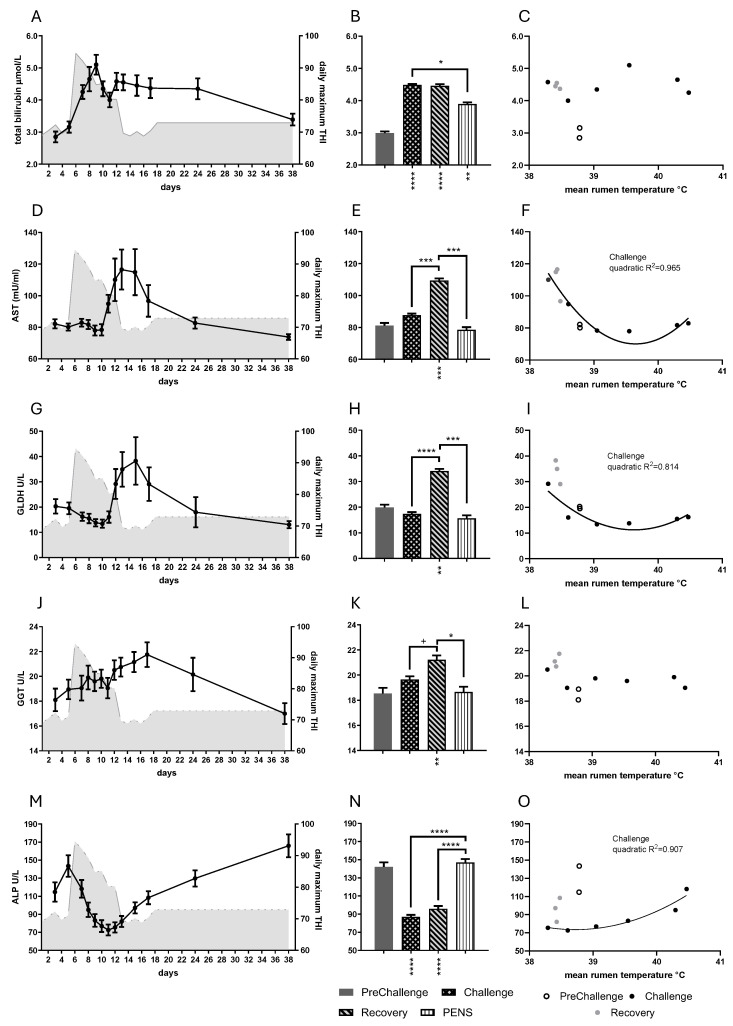
Behavior of total bilirubin concentration and the liver enzyme activities, ALP, AST, GLDH and GGT. Panels (**A**,**D**,**G**,**J**,**M**): Trajectories of the daily mean (±SE) of each analyte over the 38-day trial underlaid with the daily maximum THI profile (shaded area). Note that the line connecting the data points is interpolated. Panels (**B**,**E**,**H**,**K**,**N**): Histograms of the period means (±SE) of each analyte during PreChallenge, Challenge, Recovery and PENs. Significant differences with the PreChallenge mean are indicated by asterisks below the *x*-axis. Significant differences amongst the later periods are denoted by asterisks above the brackets. +, *p* < 0.10; *, *p* < 0.05; **, *p* < 0.01; ***, *p* < 0.001; ****, *p* < 0.0001. Panels (**C**,**F**,**I**,**L**,**O**): Plots of the relationships of the daily mean of each analyte with rumen temperature. The coefficient of determination (R^2^) is displayed where a quadratic relationship was found.

Period significantly influenced the behavior of the plasma indicators of liver function: total bilirubin concentration and the enzyme activities of ALP, GLDH, AST and GGT (*p* < 0.0001 in all cases; Figure 5). Daily mean total bilirubin concentration rose rapidly with the onset of HHL, peaking on day 9. It fell briefly and curiously, peaked a second time on day 12, and remained high for the remainder of Recovery and into PENs (Figure 5A). All the period means were greater than the PreChallenge mean (Figure 5B, Table 1). Daily mean total bilirubin concentration showed no relationship with core temperatures (Figure 5C, Supplementary Figure S7A).

The enzymes, except for ALP, all showed maximum activity during Recovery, as opposed to Challenge. Daily mean AST activity was very stable for much of the Challenge and similar to PreChallenge levels until day 11. It rose rapidly and came to a plateau on days 13 and 15 (Recovery) and then fell (Figure 5D). The mean in Recovery was greater than the PreChallenge mean (Figure 5E, Table 1). Daily mean GLDH activity followed a similar trajectory (Figure 5G). GLDH activity was stable and even slightly reduced during much of the Challenge, rising on day 12, and peaking on day 15. The mean in Recovery was greater than the PreChallenge mean (Figure 5H, Table 1). Both enzyme activities showed high variability throughout Recovery and into PENs. Daily mean AST and GLDH activities exhibited strongly correlated quadratic relationships with RumT during Challenge only (Figure 5F,I).

Daily mean GGT activity increased over the first three periods. GGT activity came to a maximum on day 17, the last day of Recovery when RumT was low (Figure 5J,L). The Recovery mean was greater than all other period means (Figure 5K, Table 1). No relationships were detected between mean daily GGT activity and the core temperatures (Figure 5L, Supplementary Figure S7D). Unlike the other enzymes, daily mean ALP activity fell precipitously with the onset of HHL, falling to 72.6 U/L on day 11, and then rising gradually through Recovery and PENs (Figure 5M). The period means for Challenge and Recovery were less than the PreChallenge mean (Figure 5N, Table 1). Mean daily ALP activity underwent a quadratic response to changing core temperatures in Challenge (Figure 5O; Supplementary Figure S7E).

The activities of AST and GLDH were strongly positively correlated (Supplementary Figure S8A). Daily mean total bilirubin concentration was strongly negatively correlated with cholesterol concentration (Supplementary Figure S8B). Daily mean ALP activity presented strong linear relationships with the concentrations of the energy metabolites, cholesterol, glutamine and BOHB (Supplementary Figure S8C–E). Of the interactions with the metabolic hormones, total bilirubin concentration was strongly negatively correlated with T4 and leptin (log10) concentrations (Supplementary Figure S9A,B). Daily mean GGT activity returned a negative correlation and linear relationship with insulin concentration (Supplementary Figure S9C). AST, GLDH and ALP activities were not correlated to the concentrations of the metabolic hormones.

### 3.4. Buffering and Electrolytes

**Figure 6:**
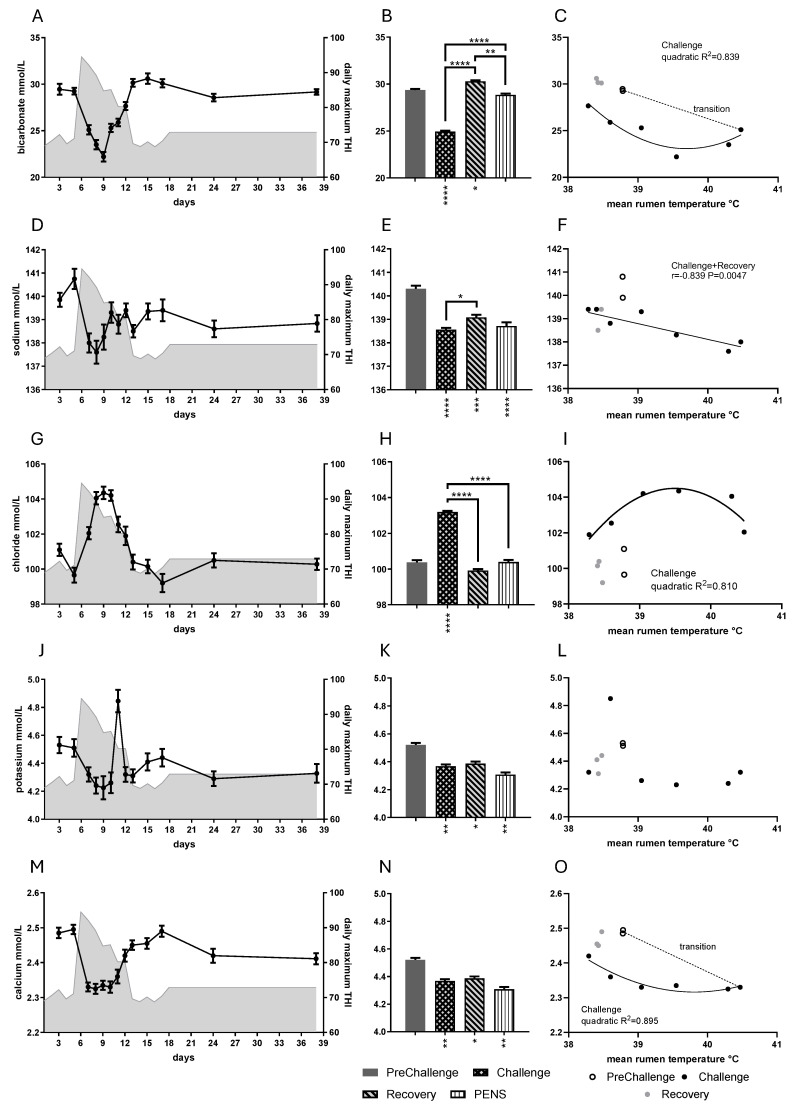
Behavior of bicarbonate concentration and electrolyte concentrations, sodium, chloride, potassium and calcium. Panels (**A**,**D**,**G**,**J**,**M**): Trajectories of the daily mean (±SE) of each analyte over the 38-day trial underlaid with the daily maximum THI profile (shaded area). Note that the line connecting the data points is interpolated. Panels (**B**,**E**,**H**,**K**,**N**): Histograms of the period means (±SE) of each analyte during PreChallenge, Challenge, Recovery and PENs. Significant differences with the PreChallenge mean are indicated by asterisks below the *x*-axis. Significant differences amongst the later periods are denoted by asterisks above the brackets. *, *p* < 0.05; **, *p* < 0.01; ***, *p* < 0.001; ****, *p* < 0.0001. Panels (**C**,**F**,**I**,**L**,**O**): Plots of the relationships of the daily mean of each analyte with rumen temperature. Where applicable, the transition from the PreChallenge mean to the first day of Challenge is indicated (dotted line). The coefficient of determination (R^2^) is displayed where a quadratic relationship was found.

Bicarbonate and the electrolyte concentration all responded to HHL, but the magnitudes and directions of change differed. Significant period-based differences were observed for the concentrations of bicarbonate, sodium, chloride, and calcium (*p* < 0.0001; Figure 6). Daily mean bicarbonate concentration fell rapidly with the onset of heat load, arriving at its minimum (22.2 mM) on day 9. It recovered quickly, so that by day 13 the concentration was close to PreChallenge levels (Figure 6A). The Challenge mean was less than the PreChallenge mean, but the Recovery mean showed a slight “overshoot”; it was 3.2% greater than the PreChallenge mean (Figure 6B, Table 1). Daily mean bicarbonate concentration possessed strongly correlated quadratic relationships with core temperatures during Challenge (Figure 6C, Supplementary Figure S10A).

Daily mean sodium concentration decreased rapidly, coming to a minimum on day 8 (137.6 mM). The mean concentration increased on day 10 as conditions improved, and a tight range was maintained for the rest of the experiment (Figure 6D). All period means were 0.9–1.2% less than the PreChallenge mean (Figure 6E, Table 1). During Challenge and Recovery, daily mean sodium concentration showed strongly correlated negative linear relationships with core temperatures (Figure 6F; Supplementary Figure S10B). Daily mean chloride concentration rose rapidly to come to a plateau of ~104 mM over days 8–10, and fell as quickly to stabilize in PENs (Figure 6G). The mean concentration in Challenge was 2.8% greater than the PreChallenge mean (Figure 6H, Table 1). The means in Recovery and PENs did not differ from the PreChallenge mean. Quadratic relationships (inverted parabolas) with core temperatures were evident during Challenge (Figure 6I; Supplementary Figure S10C).

Daily mean potassium concentration initially fell to 4.22–4.26 mM on days 8–10. There was an abrupt peak on day 11 (4.85 mM) experienced by most animals (Figure 6J). The effect of period on potassium was less (*p* = 0.0115; Figure 6K). All the period means were 3.0–4.7% less than the PreChallenge mean (Figure 6K). There were no detectable relationships between the core temperatures and daily mean potassium concentration (Figure 6L, Supplementary Figure S10D). Daily mean calcium concentration fell immediately as conditions warmed (Figure 6M) and remained low and stable for most of Challenge. It rose steadily from day 12, peaking on day 17. The Challenge and PEN means were less than the PreChallenge mean r (Figure 6N, Table 1). There was a strongly correlated quadratic relationship between daily mean calcium concentration and RumT during Challenge (Figure 6O), which was not evident with RecT (Supplementary Figure S10E).

There were notable cross-correlations amongst the electrolytes and with bicarbonate (Supplementary Tables S2 and S3, Supplementary Figures S11 and S12). Bicarbonate concentration showed strongly correlated linear but opposing relationships with chloride concentration (Supplementary Figure S11A) and calcium concentration (Supplementary Figure S11B). Sodium concentration was positively and linearly correlated with calcium concentration (Supplementary Figure S11F), whereas chloride concentration was negatively correlated with calcium concentration (Supplementary Figure S12A). The ion concentrations also produced strongly correlated linear relationships with creatinine concentration (Supplementary Tables S2 and S3). Creatinine concentration was negatively correlated with the concentrations of bicarbonate (Supplementary Figure S11C), sodium (Supplementary Figure S11G), and calcium (Supplementary Figure S12D). In the case of calcium and creatinine concentrations, a quadratic model was also credible (Supplementary Figure S12D). Urea concentration was also negatively correlated with bicarbonate concentration (Supplementary Figure S11E). Interestingly, sigmoidal curves were more appropriate fits for the relationships between bicarbonate concentration and creatinine and urea concentrations (Supplementary Figure S11C,E). Chloride concentration was positively correlated with creatinine concentration (Supplementary Figure S12B) but negatively correlated with glutamine concentration (Supplementary Figure S12C). Bicarbonate concentration returned a positive correlation with GLDH activity in Challenge and Recovery (Supplementary Figure S11D). Only one hormone, T4, returned relationships with the electrolytes. T4 concentration was positively and linearly correlated with bicarbonate, sodium, and calcium concentrations (Supplementary Figure S13A–C).

### 3.5. Total Protein and Albumin

**Figure 7:**
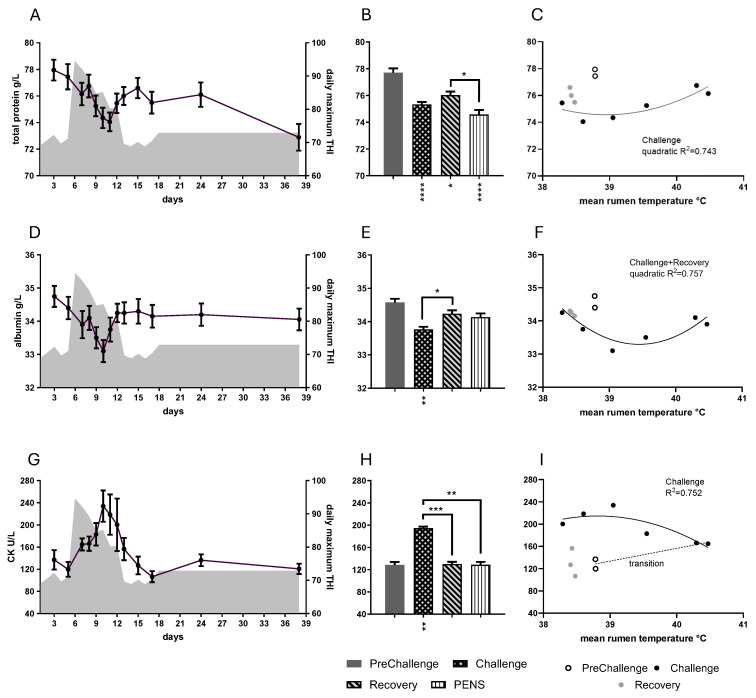
Behavior of total protein and albumin concentrations and CK activity. Panels (**A**,**D**,**G**): Trajectories of the daily mean (±SE) of each analyte over the 38-day trial underlaid with the daily maximum THI profile (shaded area). Note that the line connecting the data points is interpolated. Panels (**B**,**E**,**H**): Histograms of the period means (±SE) of each analyte during PreChallenge, Challenge, Recovery and PENs. Significant differences with the PreChallenge mean are indicated by asterisks below the *x*-axis. Significant differences amongst the later periods are denoted by asterisks above the brackets. *, *p* < 0.05; **, *p* < 0.01; ***, *p* < 0.001; ****, *p* < 0.0001. Panels (**C**,**F**,**I**): Plots of the relationships of the daily mean of each analyte with rumen temperature. Where applicable, the transition from the PreChallenge mean to the first day of Challenge is indicated (dotted line). The coefficient of determination (R^2^) is displayed where a quadratic relationship was found.

Period influenced the concentrations of total protein (*p* = 0.0010), globulin (*p* = 0.0020), and albumin (*p* = 0.0054; Figure 7). Daily mean total protein concentration fell gradually during most of the Challenge period, achieving a minimum on day 11 (Figure 7A). It increased over the following four days to peak on day 15. The Challenge and PEN means were less than the PreChallenge mean, respectively (Figure 7B, Table 1). Daily mean total protein concentration showed a strong quadratic relationship with RumT during Challenge (Figure 7C), whereas a linear relationship was evident with RecT (Supplementary Figure S14A). As the globulin concentration followed that of total protein (Supplementary Figure S15A), the 38-day trajectory, period comparisons and interaction with RumT are given in Supplementary Figure S15B–D.

Daily mean albumin concentration reduced to reach a minimum on day 10 (Figure 7D). By day 12, it was close to PreChallenge levels and stayed within a narrow range for the remainder of the trial. The mean concentration during Challenge was less than the PreChallenge mean (Figure 7E, Table 1). The means for the remaining two periods were not different from the PreChallenge mean. In Challenge and Recovery, daily mean albumin concentration showed a ‘shallow’ parabolic relationship with RumT (Figure 7F), whereas there was no simple relationship detected with RecT (Supplementary Figure S14B). Total protein concentration correlated with albumin concentration while in the CCR (Supplementary Figure S16A). Albumin concentration and calcium concentrations were positively correlated (Supplementary Figure S16B). Adiponectin concentration was positively correlated with total protein and albumin concentration (Supplementary Figure S16C,D). Albumin concentration was also correlated with T4 concentration (Supplementary Figure S16E).

### 3.6. Creatine Kinase (CK)

CK activity was strongly influenced by period (*p* < 0.0001). There was a staggered rise in CK activity during Challenge, peaking on day 10 and falling through Recovery (Figure 7G). The Challenge mean was greater than the PreChallenge mean (Figure 7H). Daily mean CK activity presented a ‘shallow’ inverted parabolic relationship with RumT during Challenge (Figure 7I), whereas a linear relationship was the better fit with RecT (Supplementary Figure S14C). CK activity correlated with several metabolites. Negative linear relationships were discovered with glutamine and -glucose (Supplementary Figure S17A,B). Positive linear relationships were detected with BOHB (and chloride concentrations (Supplementary Figure S17C,D). No simple relationships were found between CK activity and any of the metabolic hormones.

## 4. Discussion

**Figure 8:**
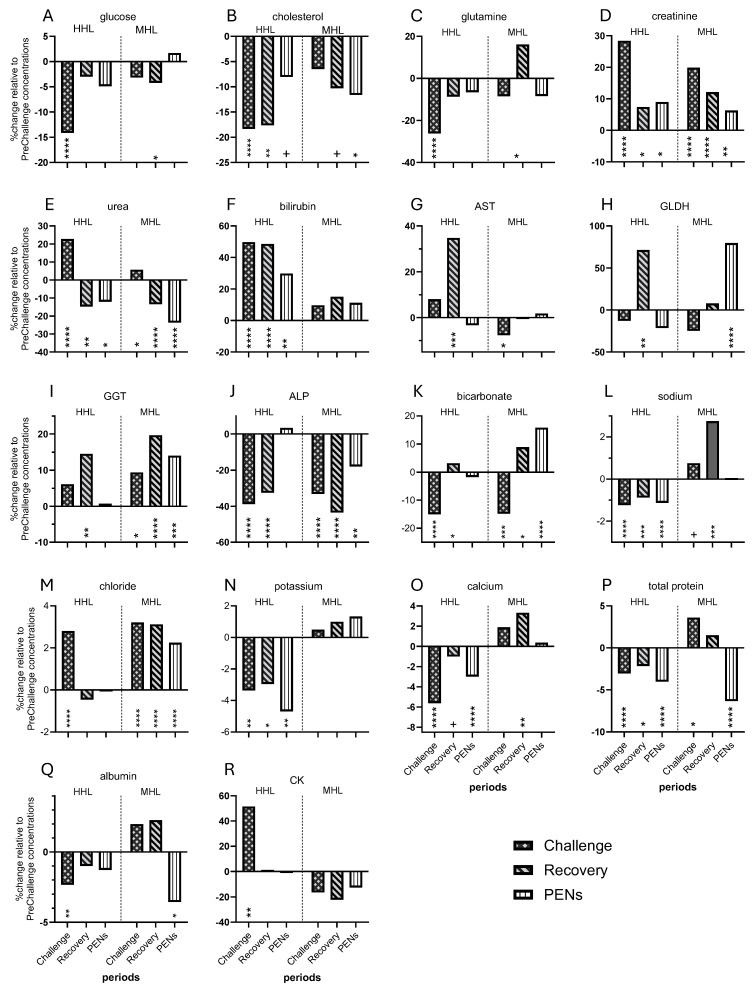
Comparison of the analyte responses over the Challenge, Recovery and PENs periods relative to PreChallenge in the current high heat load experiment (HHL) and the earlier moderate head load (MHL) experiment [10]. The period means are presented as % change from the PreChallenge mean in each case: (**A**) glucose, (**B**) cholesterol, (**C**) glutamine, (**D**) creatinine, (**E**) urea, (**F**) total bilirubin, (**G**) AST, (**H**) GLDH, (**I**) GGT, (**J**) ALP, (**K**) bicarbonate, (**L**) sodium, (**M**) chloride, (**N**) potassium, (**O**) calcium, (**P**) total protein, (**Q**) albumin, and (**R**) CK. The level of significance from the PreChallenge mean is denoted by the asterisks above the *x*-axis. +, *p* < 0.10; *, *p* < 0.05; **, *p* < 0.01; ***, *p* < 0.001; ****, *p* < 0.0001.

Due to the comprehensive nature of this study, the Discussion covers the findings for the individual analytes and then integrates the findings to describe the metabolic states of the steers in Challenge, Recovery and PENs. Figure 8 was produced to enable comparison of the MHL and HHL experiments at the level of the period mean concentrations. On the whole, the steers in PreChallenge in both experiments were in a similar baseline metabolic state (Supplementary Text S1).

### 4.1. Energy Metabolism

The concentrations of all the energy metabolites assessed responded to the onset of HHL. Except for glucose, all presented parabolic relationships with core temperature. In part, parabolic relationships reflected the nature of the thermal challenge. The sudden imposition of very high heat load (daily maximum THI > 89 for 3 days) provoked a rapid change in metabolite concentrations, which then stabilized as core temperatures were reduced over the remaining days of Challenge. For example, glutamine and cholesterol concentrations presented strongly correlated inverted parabolic relationships during Challenge. After the initial fall in concentration with increasing RumT, their concentrations came to a plateau even though RumT fell below 39.55 °C. The observation that the concentrations did not alter concurrently with RumT as conditions cooled indicated a delayed response in the organs and tissues, as reported by plasma concentrations at least.

Glucose concentration was markedly reduced during thermal challenge and rapidly rebounded to PreChallenge levels by day 13, the first day of Recovery (thermoneutral conditions). As alluded to above, there was no association with core temperature. Nor were there any relationships of glucose concentration with the metabolic hormones, including insulin. Under the conditions of this experiment, the regulation of glucose concentration appeared to be dependent on many inputs. In the MHL experiment, glucose concentration was maintained throughout the trial and moderately correlated in a linear manner with DMI in both Thermally Challenged (TC) and FRTN treatment groups. An assortment of glucose responses to thermal challenge in ruminants has been reported [23,24,25,26,27,28], most likely a consequence of differing experimental designs and housing, animal diet, age, breed, previous exposure to heat stress, dairy vs. beef cattle, etc.

Plasma BOBH concentration rose late in the thermal challenge and fell in Recovery. Plasma BOHB concentrations have not often been measured in beef cattle heat stress studies. In the lactating dairy cow exposed to brief periods of seasonal heat stress, raised BOHB plasma concentrations are reported regardless of lactation period [29,30,31]. On the other hand, Pontiggia et al. (2023) [32] found no relationship between vaginal temperature and plasma BOHB concentration of lactating cows during repetitive bouts of seasonal heat stress. Also, Lamp et al. (2015) [33] could not differentiate the BOHB response in pair-fed thermoneutral (PFTN) or heat-stressed cows, whether ante-partum or post-partum. Unfortunately, BOHB was not measured in the MHL experiment, and comparison cannot be made with the current study. However, BOHB concentration is a dependable indicator of fatty acid mobilization and fatty acid oxidation [34]. Variable non-esterified fatty acid (NEFA) responses, no change or increased NEFA plasma concentrations, have been reported regardless of cohort and/or comparison to PFTN controls [24,25,27,28,30,31,32]. In the MHL experiment, there was no effect of heat stress on NEFA concentration [10].

The HHL experiment engendered a prolonged deficit in cholesterol concentration throughout the latter three periods (8–18%). Reduction in cholesterol concentrations as a direct response to heat load in ruminants has been reported over decades [35,36,37]. Bobek and Ginter (1966) [38] demonstrated reduced cholesterol synthesis in heat-stressed rats. Comparing the behaviors of cholesterol concentration in the two heat load experiments further revealed the differing dynamics. While there was no change to cholesterol concentration during Challenge in the MHL experiment, there were reductions during Recovery (~10%) and in PENs (~12%) as compensatory growth was initiated (Figure 8B). Furthermore, plasma cholesterol concentration was not associated with core temperatures in the TC group (MHL experiment). Thus, the severity of thermal challenge influences the cholesterol response.

Glutamine is a critical energy substrate in many tissues. Plasma glutamine concentration fell rapidly with the onset of HHL and eventually returned to PreChallenge levels on the last day of Recovery, contrasting with glucose concentration, which arrived at PreChallenge concentration on the first day of Recovery. The two reports on glutamine concentration in heat-stressed dairy cows gave variable responses, with no change [39] or decreased concentration [40]. Glutamine concentration was unchanged by thermal challenge in the MHL experiment, whereas there was a 26% deficit seen during the HHL Challenge (Figure 8C). Furthermore, the HHL experiment could not replicate the 16% rise in Recovery observed in the MHL trial. Glutamine concentration in the MHL experiment appeared to be responsive to core temperatures also. There was a linear relationship evident on combining the data of both treatment groups (r = 0.895, *p* < 0.0001; Supplementary Figure S18A). Thus, during the MHL experiment, glutamine concentration reacted immediately and in a linear fashion to changing core temperatures. This was clearly not the case in the HHL experiment.

### 4.2. Creatinine and Urea

Creatine is produced by the liver and circulated in the blood to be actively transported and stored in skeletal muscle. However, there is evidence that skeletal muscle is capable of substantial creatine production given its mass in most mammals [41,42]. Within the muscle, 1–2% of the creatine is converted to creatinine, which diffuses freely from myocytes into the blood [43]. As a waste product, creatinine is freely filtered out to the circulation by the kidney; thus, elevated plasma creatinine concentrations have long been used as a primary indicator of kidney performance and injury. High levels of plasma creatinine may also occur with high muscle mass and rhabdomyolysis [43].

Plasma creatinine concentration increased rapidly with the onset of thermal challenge and subsided as soon as conditions cooled. Transient increases in plasma creatinine concentration are frequently observed in studies of heat stress in production animals [26,33,44,45,46], probably reflecting the substantial decrease in renal blood flow [47]. A solid body of work has shown reduced renal flow during passive heat stress in many species (reviewed [43]. Plasma creatinine was very sensitive to rising core temperature with an almost immediate response. In the HHL experiment, the creatinine concentration response to changing RumT during Challenge and Recovery could be described as a positive linear or a quadratic (inverted parabolic) relationship. Similarly, a positive linear relationship was found between creatinine concentration and core temperature in the MHL experiment (r = 0.877, *p* = 0.0095; Supplementary Figure S18B).

That the greater thermal load in HHL provoked greater elevation in plasma creatinine during Challenge than that seen in the MHL Challenge was not surprising; however, the differing behaviors in Recovery and PENs periods need some interpretation (Figure 8D). Plasma creatinine concentration fell quickly in the HHL experiment, whereas it was reduced but remained significantly elevated in later periods in the MHL experiment. It is unlikely that creatinine clearance was restored more rapidly in the HHL steers than the MHL steers in Recovery. A possible explanation is that the MHL steers were already embarked on an anabolic trajectory in Recovery [11], whereas the HHL steers had not [17]. Thus, in the MHL scenario, skeletal muscle repair and growth were promoted with subsequent release of creatinine, contributing to raised plasma creatinine concentration.

Similar to plasma creatinine, urea concentration increased rapidly with thermal load and fell as quickly in cooling conditions and before thermoneutral conditions were imposed in Recovery. In the current study, urea concentration showed biphasic responses as core temperatures fell in Challenge, reflecting the lag in lowering the urea concentration. After the initial rise in core temperature, urea concentration stabilized and then reduced at an exponential rate. In the kidney, approximately half of the excreted urea is resorbed along with water. Like creatinine, reduced renal blood flow and the GFR impacted its excretion with subsequent changes to the plasma concentration. The HHL challenge provoked a uremic state. This has been frequently observed in heat-stressed dairy cattle [26,27,48,49] but not so in bulls and beef cattle [23,24,50]. In concordance with the latter studies, the MHL experiment did not exhibit higher urea concentrations in Challenge relative to the FRTN steers (Figure 8E, ref. [10]).

The urea trajectory in the HHL experiment differed from the linear response with core temperatures obtained in the MHL experiment (Supplementary Figure S18C). Thus, following the HHL challenge, there was not an immediate homeorhetic response as seen in the MHL experiment. The HHL Recovery and PENs did not exhibit the deficits in urea concentration seen in the MHL experiment. It is well known that plasma urea concentration decreases with re-alimentation and compensatory growth [51,52,53]. The smaller deficit seen in PENs in HHL as compared with MHL PENs has been interpreted as delayed anabolism/compensatory growth in the HHL steers.

### 4.3. Liver Function

The liver function biomarkers responded strongly to the HHL challenge. The most distinctive of these was the total bilirubin concentration, which persisted at 30–50% greater than the PreChallenge concentration over the latter three periods. Not surprisingly, it showed no relationships with core temperatures, nor most of the metabolites or enzymes. Bilirubin concentration was not altered in the MHL experiment (Figure 8F, ref. [10]), indicating the profound change in liver function that occurs in HHL. Despite its ubiquity in clinical biochemistry panels, plasma total bilirubin concentration has rarely been measured in heat stress studies until recently. Raised bilirubin levels have been detected in heat-stressed lactating dairy cows [31,54] and gilts [55].

AST and GLDH activities were unchanged during thermal challenge but were elevated in Recovery. The trajectories of AST and GLDH were very similar despite AST’s broader tissue distribution and less specificity for hepatic cellular damage [56]. During the hottest conditions of Challenge, the daily activity levels of the two enzymes remained within a tight range and were equivalent to the PreChallenge level or lower. Both enzyme activities increased abruptly in late Challenge to attain maximal plasma activity on day 15 during Recovery, and there was large inter-animal variation. The MHL experiment also revealed unchanged to low plasma activities in Challenge, and high activity in Recovery and PENs in the TC treatment groups (Figure 8G,H, ref. [10]). In the current experiment, the enzyme activities revealed standard quadratic relationships with core temperatures, with the greatest plasma activity coinciding with hypothermic/low normothermic core temperatures in Recovery.

Superficially, GGT activity was unchanged during challenge and rose in Recovery. However, the trajectory for GGT activity differed from those of AST and GLDH. Firstly, there was a small rise in Challenge. Secondly, GGT activity apparently achieved maximum activity on day 17, but given that the next data point was obtained on day 24, the trajectory over the intervening days is unknown. Its behavior was independent of core temperatures. Long considered a ‘liver’ enzyme, its raised levels in diverse chronic medical conditions have seen plasma GGT activity touted as a marker of systemic oxidative stress [57,58]. Furthermore, plasma GGT activity has been negatively associated with insulin resistance and insulin concentration in healthy adults and diabetic patients [59,60]. Accordingly, we detected a strong negative correlation between GTT activity and insulin levels in this trial. It is intriguing to observe that the HHL steers, when compared to the TC and FRTN steers of the MHL experiment, had the least GGT response to thermal challenge (Figure 8I). Most notably, during PENs, the HHL steers returned to PreChallenge GGT activity, whereas it remained high in both cohorts of the MHL experiment [10]. These two cohorts, with rapid weight gain, appeared to be on the verge of insulin resistance based on their high insulin and glucose levels [11].

Plasma ALP activity was substantially decreased in Challenge and Recovery. Large reductions in plasma ALP activity occurred in Challenge, which was highly reminiscent of ALP activity in the MHL experiment (Figure 8J). Falls in plasma ALP activity have been reported for lactating dairy cows [37] and sheep [61], although Blond et al. (2024) [31] found no change regardless of lactation period and level of heat load. It seems that heat stress in ruminants, at least, is distinctive in that this ‘rule’ is not followed. Most ALP in adult plasma is composed equally of hepatic and bone glyco-isoforms [62]. Assay of plasma bone ALP (BALP) activity from this experiment showed that BALP activity was approximately 50% lower than PreChallenge levels during much of Challenge ([63], Supplementary Text S2). This result, as well as plasma markers of bone formation and resorption, was suggestive of altered bone development in these young animals.

Raised plasma ALP is a marker for biliary cholestasis, and in most cases of hepatic injury, ALP activity in plasma follows the course of AST and GLDH activity. Low plasma ALP (hypophosphatasia) in the human population is rare and usually associated with mutations of the ALP genes [64]. In a retrospective study of cases of non-genetic hypophosphatasia, Hoff et al. (2025) [65] found that approximately 25% of the study population had chronic hypothyroidism. There was no correlation between the plasma T3 and T4 hormone concentrations and ALP activity in the HHL steers during the experiment. Recently, low plasma ALP activity has been described in patients with chronic liver disease [66]. The ALP levels in the steers were correlated positively with cholesterol concentrations and negatively with bilirubin concentrations. It is possible that the low ALP activity was another indicator of hepatic duress.

### 4.4. Buffering and Electrolytes

Plasma bicarbonate concentration was low during Challenge but recovered as conditions cooled. Plasma chloride concentration was elevated in Challenge and returned to PreChallenge levels in Recovery. The low bicarbonate and high chloride concentrations were indicative of a compensated respiratory alkalosis during Challenge [67]. This condition is a direct manifestation of the high respiration rates (RR) induced by heat load, the most obvious symptom of heat stress in ruminants. An almost identical bicarbonate response was seen in the MHL experiment (Figure 8K). In the HHL experiment, there was a slight but significant increase in bicarbonate concentration, and a small fall in chloride concentration in Recovery when core temperatures and RR were at their lowest. This was likely indicative of another change in acid-base balance. In PENs, the bicarbonate and chloride levels returned to PreChallenge levels. In the MHL experiment, both bicarbonate and chloride levels were higher than the PreChallenge levels in both the TC and FRTN groups, suggestive of a complex acid-base disturbance during realimentation and compensatory growth (Figure 8K,M).

The concentrations of sodium and potassium were in deficit from Challenge through to PENs. This behavior differed from the MHL experiment during which potassium concentration rose slightly (although not significantly), and sodium concentration was raised during Challenge and Recovery in the TC and FRTN groups (Figure 8L,N). The loss of the cations from the blood in the HHL Challenge may be explainable as loss to sweat and the large increase in water intake. However, the persistent deficits in Recovery and PENs remain an enigma. An intensive study of the blood acid-base balance informed by blood pH and ppCO_2_ in such an experiment would be highly instructive and may also go some way to explaining the inverted parabolic relationships between core temperature and chloride concentration.

The behavior of the plasma potassium was unique, with the conspicuous spike on day 11. This transient hyperkalaemia coincided with the first day that core temperatures returned to the normal range after thermal challenge (RumT: 38.60 °C; ref. [16]). Hyperkalaemic spikes are known to occur with reperfusion of organs and tissues following an ischemic event and are thought to be due to the release of extracellular potassium accumulated as a cellular response to hypoxia [68,69]. Calcium concentration was reduced from Challenge to PENs (Figure 8O). Ionized calcium concentration falls during an alkalosis as hydrogen ions released from albumin allow calcium ions to bind [70]. However, the calcium data reported herein refer to total calcium concentration, that is, both the ionized and the protein-bound calcium. Calcium concentration correlated with albumin concentration, but not during Challenge. For the most part, reduced albumin concentration may be responsible for the low calcium concentration. Both were correlated with T4 concentration.

The bicarbonate concentration showed strong parabolic relationships with the core temperatures during Challenge (R^2^ ~0.78 and 0.84), which differed from the negative linear response detected in the MHL experiment (Supplementary Figure S17D). On the other hand, sodium concentration presented strong linear relationships with core temperatures (r~−0.73 and 0.84), and a similar relationship occurred in the MHL experiment (Supplementary Figure S17D). Bicarbonate concentration showed strongly correlated sigmoidal relationships with urea and creatinine concentrations, revealing two different states in the relationships and the rapid transition between those states (Supplementary Figure S10C,E). When bicarbonate concentration was <25 mM, urea and creatinine concentrations were high and stable. This occurred in the most extreme conditions of Challenge. When bicarbonate concentration was >25 mM, urea and creatinine concentrations were low and stable (PreChallenge, Recovery and PENs). All three metabolites are under strong renal control.

### 4.5. Total Protein, Albumin and CK

Total protein and albumin concentrations were slightly reduced in Challenge. Total protein concentration remained low in Recovery and PENS, whereas albumin concentration returned to PreChallenge levels in the later periods. The low concentrations occurring late in Challenge contributed evidence that the steers were well hydrated. The protein concentrations were not affected in MHL; they were reduced in PENs during realimentation and compensatory growth (Figure 8P,Q). The lowered protein concentrations in the HHL experiment could be an outcome of the reduced thyroid hormone concentration in Challenge, impacting protein synthesis in the liver. Albumin concentration was strongly and positively correlated with T4 concentration. Both total protein and albumin concentrations correlated with adiponectin concentration, but this may be a consequence of the strong correlation between the two hormones [17].

CK activity was highest in Challenge and fell in Recovery. Increased levels of CK activity in plasma are a long-standing indicator of muscle damage. A rise in plasma CK is not often reported in heat stress studies of ruminants and did not occur in the MHL experiment (Figure 8R; [10]). Similarly, Blond et al. (2024) [31] reported no change in CK activity in lactating heat-stressed dairy cows. Furthermore, Roths et al. (2023) [49] demonstrated the absence of increased calpain activity, integral to proteolysis, in skeletal muscle of heat-stressed dairy cows. However, the large reductions in blood flow to most skeletal muscles in heat-stressed ruminants [71] could set the conditions for muscle damage if hyperthermia becomes severe. In the current experiment, the increase in plasma CK appeared in two stages: a smaller rise and plateau for the most part of Challenge and then a rapid rise as conditions cooled in late Challenge and into Recovery. CK activity during this late peak was characterized by large variability, with maximum activity occurring on day 10, when RumT fell below 39.55 °C.

### 4.6. Synthesis—Challenge

During Challenge, four main actors were in play. Firstly, blood flow is redirected to the periphery away from the viscera and skeletal muscle, the altered endocrine milieu is dominated by low levels of thyroid hormones and adiponectin, respiratory alkalosis and a substantial reduction in feed intake. The end goal was to reduce endogenous heat production.

To ‘dump’ the excessive heat load, RR was raised to a mean daily maximum of 174–194 bpm with mean daily maximum panting scores of 2.7–3.2 on days 6–8 [16]. The compensated respiratory alkalosis was a direct consequence of this extreme respiratory state. DMI fell by 70% as a daily average to reduce rumen microbial fermentation [16]. The fermentative products, short-chain volatile fatty acids, supply the liver with substrates for gluconeogenesis and cholesterol synthesis. Rumen pH was significantly altered during a similar thermal challenge as applied to steers in the current experiment. In the late Challenge, rumen pH ranged between 6.6 and 6.8, indicative of reduced fermentation, and diurnal pH cycling had collapsed [72]. The other major but less observable physiological change is the altered blood flows from viscera and skeletal muscle to the skin to facilitate direct heat loss to the environment and evaporative heat loss supported by markedly increased water usage (~100 L/head/day). The consequences of this blood redistribution are numerous. The reduced blood flow to the major organs constrains endogenous heat production in many ways. The limited blood flow to the rumen results in reduced delivery of urea to the rumen, which in turn limits microbial protein synthesis and growth. Moreover, there are consequences for normal organ functions such as glomerular filtration by the kidneys and synthesis, supply and traffic of metabolites amongst the organs, of which the liver has a major role and probably localized hypoxia.

The previous study on the metabolic hormone concentrations of the steers had predicted constrained energy metabolism and protein synthesis [17]. The HHL Challenge instigated a marked reduction in the concentrations of the major energy metabolites in plasma: glucose, glutamine and cholesterol (as an integral component of lipoproteins). As the steers became hypoglycemic, BOHB concentration increased late in Challenge, indicating some availability of fatty acids, as NEFA and/or triglycerides (TGs), for oxidation. The elevation in BOHB concentration during Challenge evidenced the switch to fatty acid oxidation in the context of low glucose, glutamine and cholesterol concentrations, and most likely raised NEFA concentration. This occurred despite the apparently depressive effects of low thyroid hormones and adiponectin concentrations on hepatic fatty acid oxidation and lipolysis in adipose depots. Presumably, the fatty acids were released from adipose depots even though there is evidence that adipose tissue increases the removal of TGs from circulation for storage during heat stress [38] by pre-regulated expression of adipose lipoprotein lipases [73]. BOBH concentrations rose and came to a plateau during Challenge when DMI was reduced to 5.5–7.0 kg/head/day. With the low feed intake and low concentration of fat in the diet (5%), it is unlikely that the diet was a source of NEFA. It is possible that the actions of another hormone have overridden the effects of the low thyroid hormones and adiponectin concentrations. A likely candidate is cortisol, which was not measured in this experiment [74,75]. The endocrine responses to substantially reduced food intake are very complex [76]. By contrast, during Challenge in the MHL experiment, the energy metabolites were more available to the TC steers. The animals were euglycemic, NEFA concentration rose, TG concentration was increased, and glutamine and cholesterol concentrations were not different from PreChallenge (but were lower than those of the FRTN group).

In the current experiment, the appearance of BOHB seemed to be highly coordinated with the reductions in glutamine and cholesterol concentration. These metabolites were strongly correlated throughout the trial, including in Challenge. Glucose and glutamine concentrations were positively correlated. Hypoglycemic humans have reduced uptake and release of glutamine from skeletal muscle, a major source of plasma glutamine [77]. Furthermore, although we found no correlation between the concentrations of the thyroid hormones and glutamine, low levels of T3 and T4 suppress glutamine release from the skeletal muscle [78].

The activities of major organs, liver, kidneys and skeletal muscle responsible for the synthesis and release of these metabolites appeared to be highly constrained for much of the HHL experiment. The low ebb in metabolic activity might be anticipated given the reduced blood flow and low levels of thyroid hormones. With the blood flow diverted from the viscera to the extremities in heat stress [47], the appearance and persistence of bilirubin were likely due to liver dysfunction caused by hepatic hypoperfusion [79]. The dampened very stable release of AST and GLDH to the plasma and the reduced concentrations of cholesterol, glutamine and glucose do not speak to a highly active or inflamed organ. Likewise, reduced total protein and albumin concentration indicate some level of hepatic dysfunction and/or a part of the hepatic response to low T4 [80].

Renal function appeared limited in the first days of Challenge as evidenced by high urea and creatinine concentrations. The reduced blood flow to the kidney and low levels of the thyroid hormones reduce GFR [81,82]. Thus, an important effector of plasma creatinine and urea concentrations was the modulation of the thyroid hormones on GFR. In the current experiment, creatinine concentration was strongly negatively correlated to T4 concentration across the trial, including in the Challenge period. As RumT fell below 39.55 °C (over days 9–10), the excess urea and creatinine were rapidly cleared from the plasma and likely to be indicative of restoration of near normal GFR by returning blood flows.

In Challenge, the steers developed a chronic respiratory alkalosis due to their very high RRs. The steers’ electrolyte status was characterized by lowered concentrations of bicarbonate, sodium and potassium, and high chloride concentrations. There was strong coordination of bicarbonate and chloride concentrations. Renal compensation involves excretion of bicarbonate and sodium, with retention of hydrogen ions and chloride (reviewed [83]). The strong coordination of bicarbonate and chloride concentrations, and their correlation with creatinine, was indicative of renal control. Reduced plasma potassium concentration frequently accompanies the electrolyte changes in chronic respiratory alkalosis [84]. Respiratory alkalosis causes vasoconstriction, reinforcing the already reduced blood flow to the organs but counteracting cutaneous vasodilation for heat loss.

### 4.7. Synthesis—Recovery and Outdoor Pens

The steers were in thermoneutral conditions in Recovery. The effectors in Recovery were low RR alongside hypothermic/low normothermic core temperatures, even though DMI had increased to approximately 75% PreChallenge intake. However, the rumen has not normalized at this stage. The rumen pH is now acidotic as fermentation rates are re-established, and diurnal pH cycling is exaggerated [52]. Blood supply was returning to tissues and organs, and a new endocrine milieu was established. The metabolic hormone profile was composed of reduced insulin concentration, and while T4 and adiponectin concentrations had improved, they remained lower than the PreChallenge means [17]. By the first day of Recovery, the steers were euglycemic, but had retained high BOHB concentration, and low glutamine and cholesterol concentrations. The lower concentration of the hormones would have constrained glucose uptake and consumption in skeletal muscle, even though reduced insulin levels would have been permissive of maintaining glucose in circulation. Fatty acid oxidation and protein synthesis should also be curtailed. In the liver, fatty acid oxidation and gluconeogenesis would also be limited. Generally, energy metabolism was constrained to some extent and may go some way to explaining the low core temperatures. The slow recovery of glutamine may speak to its requirement for gluconeogenesis and protein synthesis, but its release into plasma from the liver and skeletal muscle may still be restricted. Tissue repair and growth, and intestinal function would have been impacted by the persistently low levels of cholesterol, which is required for cell membranes and bile salt production. Urea concentration remained low also. Glutamine has a major role in trafficking ammonia arising from protein catabolism to the liver to convert to urea. The fact that plasma concentration was low may reflect low production of ammonia and little protein degradation. Furthermore, there may be conservation of N for protein synthesis. On another note, glutamine concentration was negatively correlated with CK activity; that is, there was no flood of glutamine into the plasma from damaged skeletal muscle in late Challenge and in Recovery.

While renal function seemed to have rebounded rapidly within the Challenge period, as discussed above, liver and skeletal muscle may not have returned to normal function as quickly. One interpretation of the abrupt rises in AST, GLDH and GGT activities from day 12 and the high levels in Recovery is that these were consequences of the liver undergoing a process akin to a hepatic reperfusion. In the ischemic liver, the returning blood flow facilitates the release of mitochondrial and cytosolic enzymes from damaged and dead cells into plasma [79]. Is it possible that under the right conditions, recovery from heat stress causes an ischemic reperfusion injury (IRI)? Cowled and Fitridge (2011) [85] define IRI as “… exacerbation of cellular dysfunction and death, following restoration of blood flow to previously ischaemic tissues”. Indeed, hepatic IRI has been recognized as an aftermath of classical heat stroke in humans [86].

The high liver enzyme levels and persistently high total bilirubin concentrations in Recovery are consistent with, but not diagnostic of, IRI. The hyperkalaemic spike, which occurred on day 11, aligns with IRI also [6,69]. However, diagnosis of IRI is highly contextual and dependent on the nature of the insult/intervention, the organs involved and the cohort/patient characteristics [87]. Discovery and validation of plasma biomarkers for cardiac, cerebral and hepatic IRI is an area of active research. For hepatic IRI, increased plasma lactate concentrations are indicative of liver hypoperfusion [88]. The appearance of metalloproteinases and neutrophil gelatinase-associated lipocalin (NGAL) is promising as plasma biomarkers of hepatic IRI [89]. Histological examination of the tissue would deliver a definitive diagnosis. A future study with the last days of Challenge or early Recovery as the experimental endpoint would enable tissue collection for histology and TUNEL (terminal deoxynucleotidyl transferase dUTP nick end labeling) staining to confirm the presence or otherwise of IRI. Although CK activity peaked during Challenge (day 10), its release from skeletal muscle may also be indicative of IRI within this tissue. It is likely that the timing of release and appearance of tissue damage products of varying molecular weights and cell compartments into the plasma will differ between organs, extent of return blood flows and IRI. The duration of the high bilirubin concentrations could be indicative of the time required for the liver to fully recover. Furthermore, bilirubin concentration was highly correlated with cholesterol concentration throughout the trial. Bilirubin, as an inhibitor of cholesterol synthesis [90], may be an input to the ongoing low cholesterol concentration.

Bicarbonate and chloride concentrations were restored by the first day of Recovery. However, both concentrations went into ‘overshoot’ in the subsequent days, so that there was increased bicarbonate concentration and reduced chloride concentration. This may be due to the low respiration rates during Recovery. Sodium and potassium concentrations also remained low. It is likely that the acid-base balance was not yet stabilized, and the steers were in a slightly acidotic state.

The animals were followed in outdoor pens (PENs) till day 38. DMI was eventually restored to 90% of PreChallenge DMI; however, live weight gain commenced only after day 31 [17]. Generally, the period showed that steers in were a ‘restored’ state, although the data and its interpretation are limited by the two blood samplings over that period. The higher metabolic rate was reflected by rectal temperature being slightly higher than PreChallenge [16]. Glucose and glutamine concentrations were restored, emphasizing the availability of these critical metabolites for metabolism. The high levels of liver enzyme activities were ‘washed’ out, showing that much of the hepatic insult had been overcome, considering that cholesterol and ALP levels only returned to PreChallenge levels on day 38, and the excess bilirubin in the plasma was still being cleared by the liver.

## 5. Conclusions

This study has revealed the differing metabolic responses observed between the HHL and MHL experiments, i.e., the differing stress and resultant strain on the steers under the two Challenge regimes. The MHL experiment saw the steers enlisting homeorhetic mechanisms, characterized by linear relationships of the analytes with core temperatures. By contrast, in the HHL experiment, most of the relationships were non-linear.

The HHL Challenge regime was based on an actual heatwave event, which started with three very hot days. The conditions induced remarkable coordination of altering energy metabolite profile and concentrations, and buffering and electrolyte concentrations. Moreover, GFR was reduced, and hepatic export of multiple metabolites and proteins appeared constrained. Systemically, metabolism appeared to have entered a suppressed state, which may have extended to bone and skeletal muscle, as a consequence of reduced blood flows and the hormone milieu. Hypoperfusion and localized hypoxia with cell death were a probable aftermath of the reduced blood flow. The cooler days of Challenge that followed the first three days of high heat load were highly informative of the dynamic responses to reducing heat load. As RumT fell to 39.04 °C (day 10), the rapid clearance of urea and creatinine is suggestive of returned renal blood flow and normalization of renal functions. The release of CK from skeletal muscle and the hyperkaleamic spike late in Challenge were the early indicators of the tissue damage induced by the rapid physiological and hormonal changes. These were followed by a surge of liver enzymes into the plasma in the Recovery period. The combination of events is suggestive of some level of ischemic reperfusion injury. More HHL experiments are required to investigate this. The return to thermoneutral conditions in Recovery did not deliver a ‘quick fix’. The steers were still dealing with electrolyte imbalances, limited availability of energy metabolites and urea. The steers were slightly hypothermic, and there was no live weight gain over this interval.

The MHL and HHL experiments have uncovered the highly dynamic system-wide responses to various levels of heat load in healthy young grain-fed beef steers. These are resilient animals, but maladaptive costs come with enduring high heat loads.

## Figures and Tables

**Table 1:** Comparison of the Challenge, Recovery and PENs periods with the PreChallenge period. The period means (±SD) are given for each analyte, along with the level of significant difference (*p*-values), % difference (%Δ) with the PreChallenge mean. n.s., not significantly difference.

Analyte	PreChallenge	Challenge			Recovery			PENs		
	Mean ± SD	Mean ± SD	*p*-Values	%Δ	Mean ± SD	*p*-Values	%Δ	Mean ± SD	*p*-Values	%Δ
Glucose mmol/L	4.64 ± 0.25	2.99 ± 0.23	<0.0001	−14.1	4.51 ± 0.43	n.s.	−3.0	4.42 ± 0.46	n.s	−4.9
BOBH mmol/L	0.230 ± 0.02	0.314 ± 0.02	<0.0001	36.5	0.270 ± 0.02	0.0135	17.4	0.226 ± 0.03	n.s	−1.0
Cholesterol mmol/L	3.46 ± 0.37	2.83 ± 0.35	<0.0001	−18.3	2.83 ± 0.35	<0.0001	−17.6	3.18 ± 0.38	0.0846	−8.0
Glutamine µmol/L	453.0 ± 33.2	334.5 ± 24.5	<0.0001	−26.1	413.6 ± 26.6	n.s	−8.6	423.2 ± 20.1	n.s	−6.6
Creatinine µmol/L	113.0 ± 5.1	145.1 ± 4.2	<0.0001	28.4	121.3 ± 4.5	0.0197	7.3	123.2 ± 6.2	0.0127	9.0
Urea mmol/L	4.64 ± 0.34	5.69 ± 0.41	<0.0001	22.6	3.95 ± 0.34	0.0084	−14.9	4.07 ± 0.36	0.441	−12.3
Total bilirubin µmol/L	2.99 ± 0.32	4.49 ± 0.29	<0.0001	50.1	4.46 ± 0.39	<0.0001	49.2	3.89 ± 0.30	0.0073	30.1
AST U/L	81.2 ± 10.5	87.7 ± 10.9	n.s	8.0	109.4 ± 10.4	0.0005	34.7	78.5 ± 10.5	n.s	−3.3
GLDH U/L	19.9 ± 6.6	17.4 ± 7.5	n.s	−12.5	32.1 ± 6.6	0.0056	71.4	15.6 ± 7.0	n.s	−21.6
GGT U/L	18.5 ± 2.9	19.7 ± 2.7	n.s	6.5	21.2 ± 2.7	0.0067	14.6	18.7 ± 2.5	n.s	1.0
ALP U/L	142.1 ± 32.1	86.9 ± 24.8	<0.0001	−38.8	95.9 ± 24.7	<0.0001	−32.5	146.9 ± 23.5	n.s	3.3
Bicarbonate mmol/L	29.4 ± 0.8	24.9 ± 0.8	<0.0001	−15.3	30.3 ± 0.8	0.0428	3.1	28.8 ± 0.8	n.s	−2.0
Sodium mmol/L	140.3 ± 0.9	138.6 ± 0.8	<0.0001	−1.2	139.1 ± 0.8	0.0004	−0.8	138.7 ± 1.0	<0.0001	−1.1
Chloride mmol/L	100.4 ± 0.8	103.2 ± 0.8	<0.0001	2.8	99.9 ± 0.6	n.s	−0.4	100.4 ± 0.7	n.s	0
Potassium mmol/L	4.52 ± 0.10	4.37 ± 0.14	0.0055	−3.3	4.39 ± 0.11	0.0284	−2.9	4.31 ± 0.10	0.0017	−4.6
Calcium mmol/L	2.49 ± 0.02	2.35 ± 0.02	<0.0001	−5.6	2.47 ± 0.03	0.0749	−1.0	2.42 ± 0.04	<0.0001	−3.2
Total protein g/L	77.7 ± 2.1	75.3 ± 2.0	<0.0001	−3.1	76.0 ± 2.0	0.0105	−2.2	74.6 ± 2.1	<0.0001	−4.0
Albumin g/L	34.6 ± 0.7	33.8 ± 0.8	0.0010	−2.3	34.2 ± 0.8	n.s	−1.1	34.1 ± 0.7	n.s	−1.4
CK U/L	128.4 ± 33.8	194.5 ± 30.2	0.0023	51.5	130.0 ± 32.6	n.s	1.2	128.8 ± 31.7	n.s	0.3

## Data Availability

Files (xlxs) containing clinical biochemistry data can be obtained from the journal.

## Supplementary Materials

The following supporting information can be downloaded at: https://www.mdpi.com/article/10.3390/ani16091379/s1. Supplementary Text S1: PreChallenge Baseline Comparisons of the MHL and HHL experiments. Supplementary Text S2: Summary of findings described by Anderson et al., 2018 ([63]). Table S1: The daily maximum and minimum air temperature, % relative humidity (%RH) and temperature humidity index (THI) for the 17 days in the climate-controlled rooms. Table S2. Linear relationships were detected amongst the daily mean plasma concentrations of the analytes. Table S3. Visualization of the notable correlations amongst analytes as presented in Table S2. Table S4: Linear relationships detected between the daily mean plasma concentrations of the analytes and the daily mean concentrations of the metabolic hormones. Table S5. Visualization of the notable correlations between analytes and hormone concentrations as presented in Table S4. Figure S1: Behavior of the metabolic hormone concentrations over the 38-day trial. Figure S2: Relationships of energy metabolites with rectal temperature over the 38 days of the trial. Figure S3: Linear relationships between BOHB and T3 concentrations. Figure S4. Linear relationships amongst the daily means concentrations of energy metabolites.: Figure S5: Relationships of creatinine and urea concentrations with core temperatures and each other. Figure S6: Relationships of creatinine and T4 concentrations over the 38 days of the trial. Figure S7: Relationships of total bilirubin concentration and liver enzyme activities with rectal temperature over the 38 days of the trial. Figure S8: Relationships of AST with GLDH activities, and total bilirubin concentration and ALP activity with energy metabolite concentrations over the 38 days of the trial. Figure S9: Relationships of total bilirubin concentration and GGT activity with metabolic hormone concentrations over the 38 days of the trial. Figure S10: Relationships of bicarbonate and electrolyte concentrations with rectal temperature over the 38 days of the trial. Figure S11: Relationships of bicarbonate and sodium concentrations with the concentrations of chloride, calcium, creatinine and urea, and GLDH activity over the 38 days of the trial. Figure S12: Relationships of chloride and calcium concentrations with each other, and the concentrations of creatinine and glutamine over the 38 days of the trial. Figure S13: Relationships of the concentrations of bicarbonate, sodium and calcium with T4 concentrations over the 38 days of the trial. Figure S14: Relationships of total protein and albumin concentrations and CK activity with rectal temperature over the 38 days of the trial. Figure S15: Behaviors of daily mean globulin concentration over the 38 days of the trial. Figure S16: Relationships of total protein and albumin concentrations with each other and calcium, adiponectin and T4 concentrations over the 38 days of the trial. Figure S17: Relationships of CK activity with the concentrations of glutamine, glucose, BOHB and chloride over the 38 days of the trial. Figure S18: Linear relationships of various analyte concentrations with core temperatures discovered for the MHL experiment during the CCR interval. Ref. [91] is cited in the Text S2.

## Abbreviations

The following abbreviations are used in this manuscript:

ALP	Alkaline phosphatase
AST	Aspartate aminotransferase
BALP	Bone alkaline phosphatase
BOHB	β-hydroxybutyrate
CCR	Climate-controlled rooms
CK	Creatine kinase
DMI	Dry matter intake
FRTN	Feed-restricted thermoneutral
GFR	Glomerular filtration rate
GGT	γ-glutamyl transferase
GLDH	Glutamate dehydrogenase
HHL	High heat load
IRI	Ischemic reperfusion injury
MHL	Moderate heat load
NEFA	Non-esterified fatty acids
NGAL	Neutrophil gelatinase-associated lipocalin
PFTN	Pair-fed restricted thermoneutral
RecT	Rectal temperature
RumT	Rumen temperature
RR	Respiration
T3	Triiodothyronine
T4	Thyroxine
TA	Air temperature
TC	Thermally challenged
TG	Triglycerides
THI	Temperature humidity index
TUNEL	deoxynucleotidyl transferase dUTP nick end labeling
