# Supplementary material for: Stress and Strain: Differentiating the Responses to High and Moderate Heat Loads and Subsequent Recovery in Grain-Fed Feedlot Steers—Plasma Biochemistry
Source: Animals (Basel). 2026 Apr 30;16(9):1379. doi: 10.3390/ani16091379 (PMC13162586; doi:10.3390/ani16091379)
Supplement: Supplementary file 1 [file animals-16-01379-s001.zip › CC7+8 biochem Suppl figures 2026.pptx]

## Slide 1
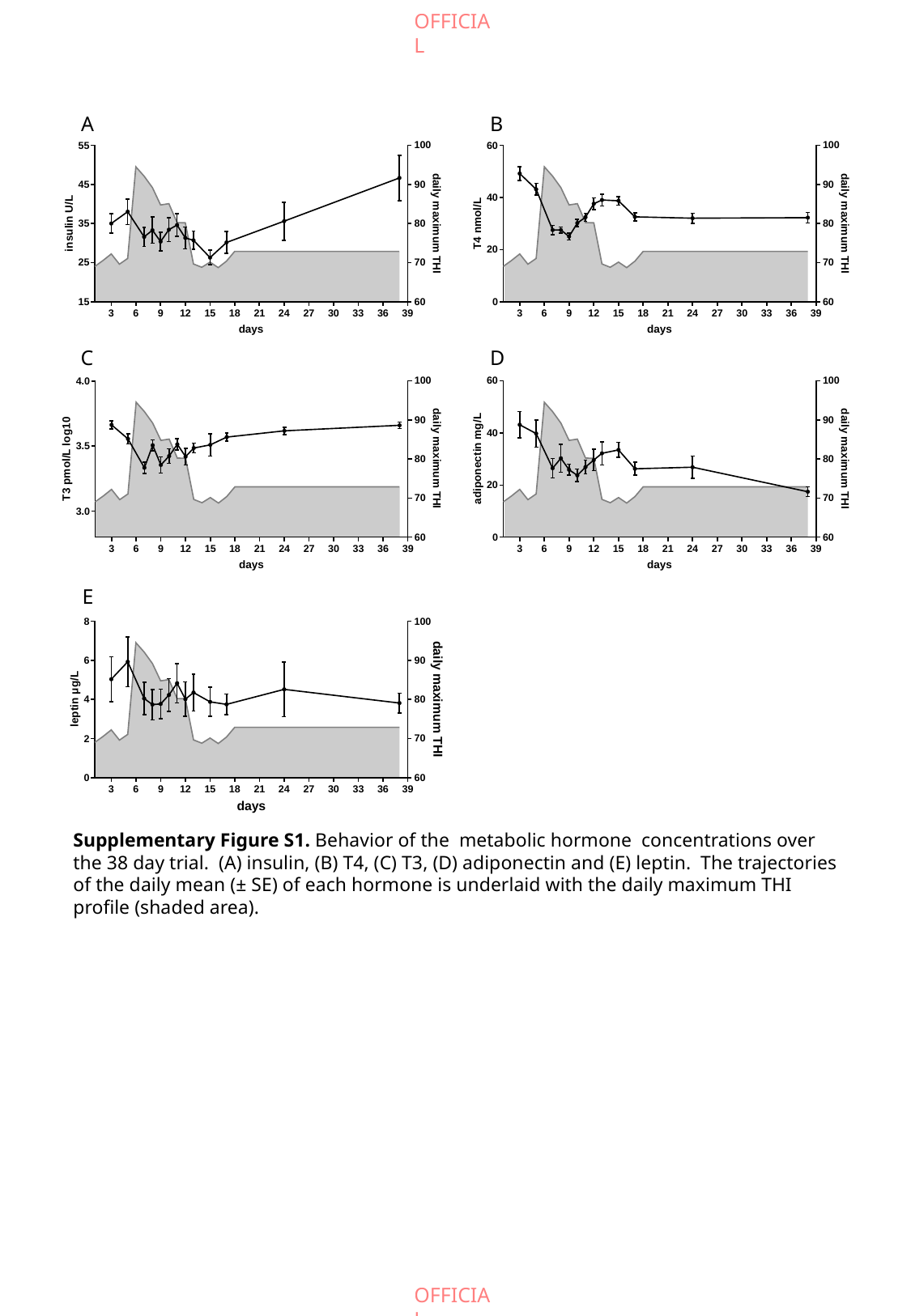

A
B
C
D
E
Supplementary Figure S1. Behavior of the metabolic hormone concentrations over the 38 day trial. (A) insulin, (B) T4, (C) T3, (D) adiponectin and (E) leptin. The trajectories of the daily mean (± SE) of each hormone is underlaid with the daily maximum THI profile (shaded area).

## Slide 2
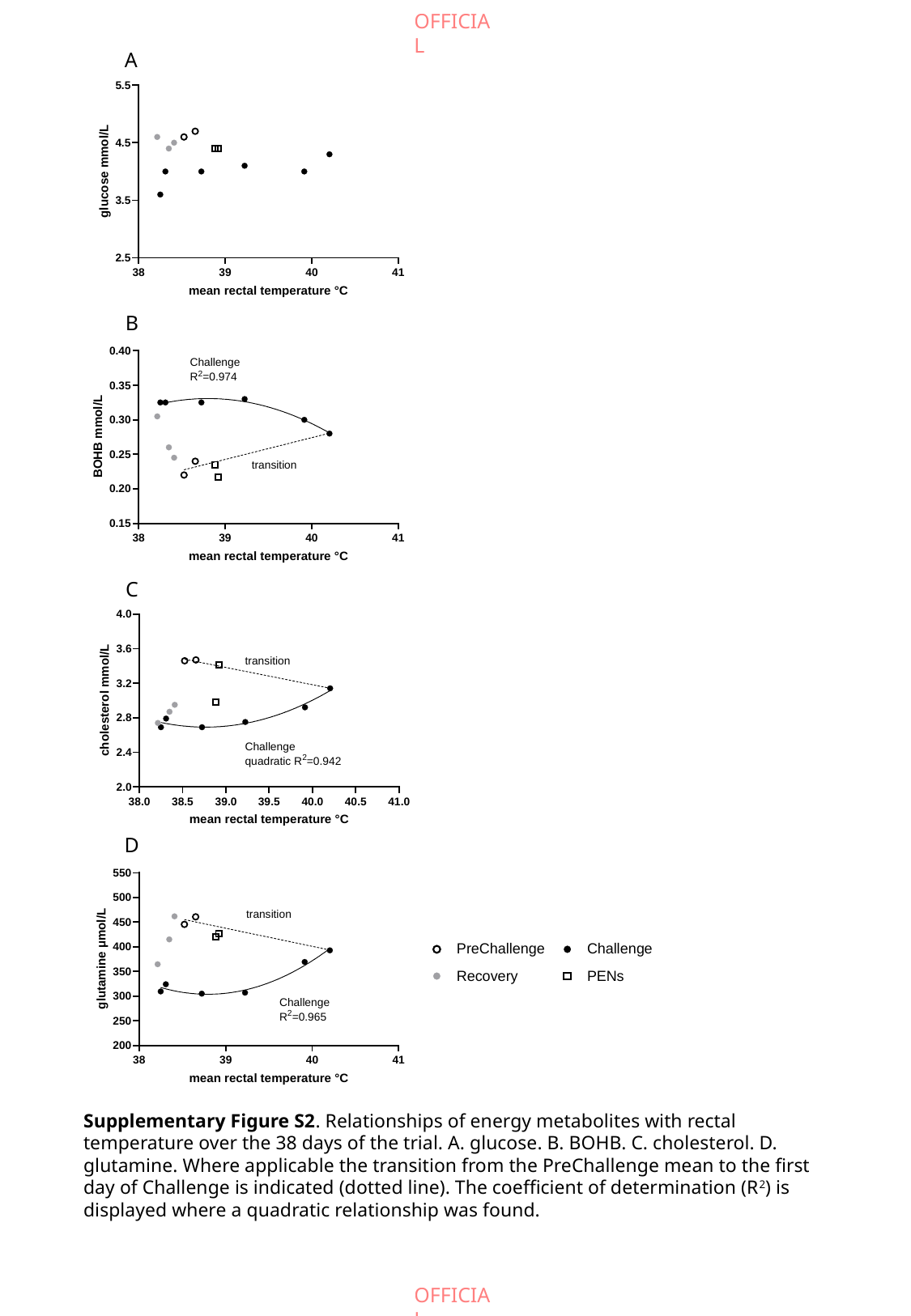

A
B
C
D
Supplementary Figure S2. Relationships of energy metabolites with rectal temperature over the 38 days of the trial. A. glucose. B. BOHB. C. cholesterol. D. glutamine. Where applicable the transition from the PreChallenge mean to the first day of Challenge is indicated (dotted line). The coefficient of determination (R2) is displayed where a quadratic relationship was found.

## Slide 3
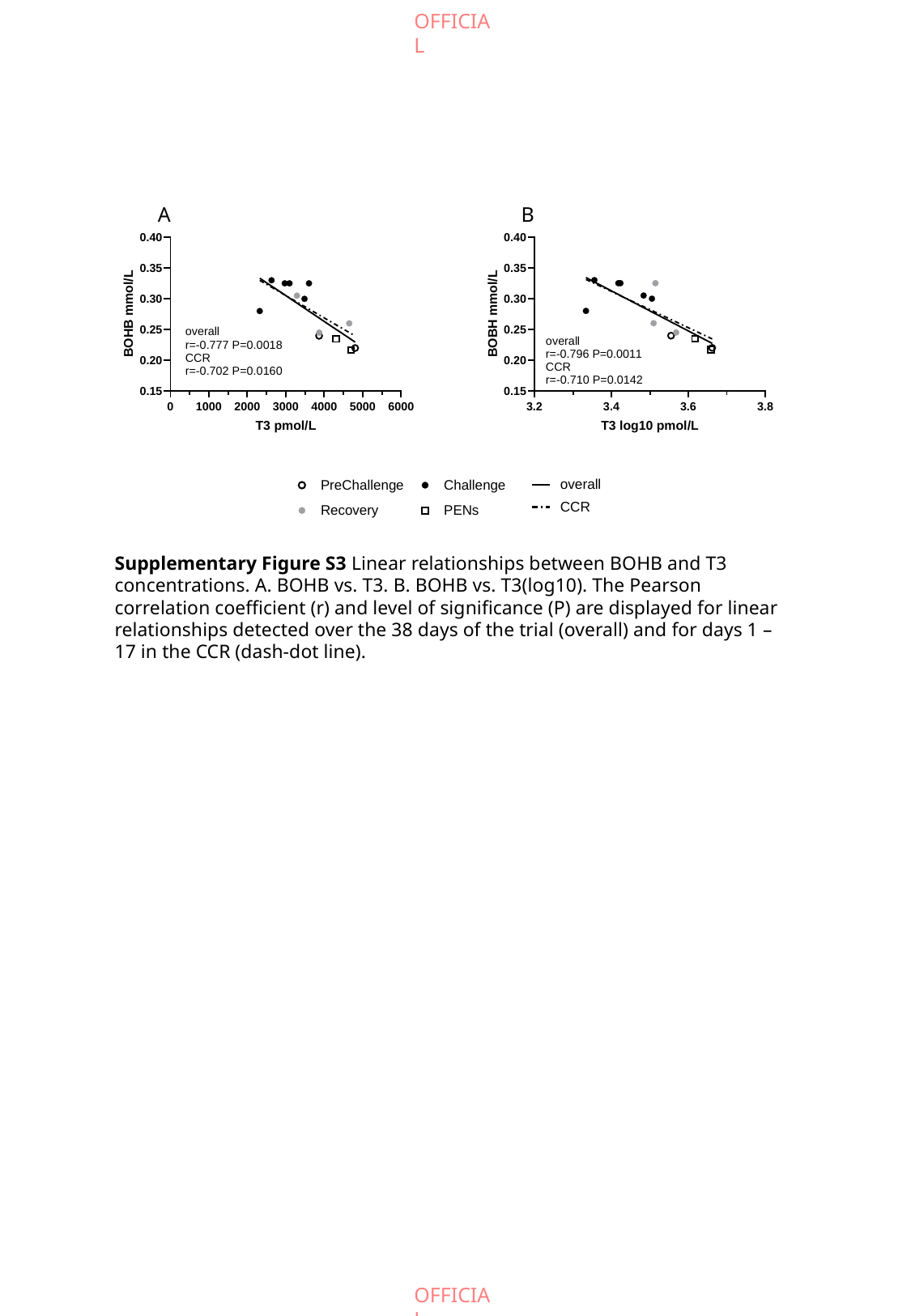

A
B
Supplementary Figure S3 Linear relationships between BOHB and T3 concentrations. A. BOHB vs. T3. B. BOHB vs. T3(log10). The Pearson correlation coefficient (r) and level of significance (P) are displayed for linear relationships detected over the 38 days of the trial (overall) and for days 1 – 17 in the CCR (dash-dot line).

## Slide 4
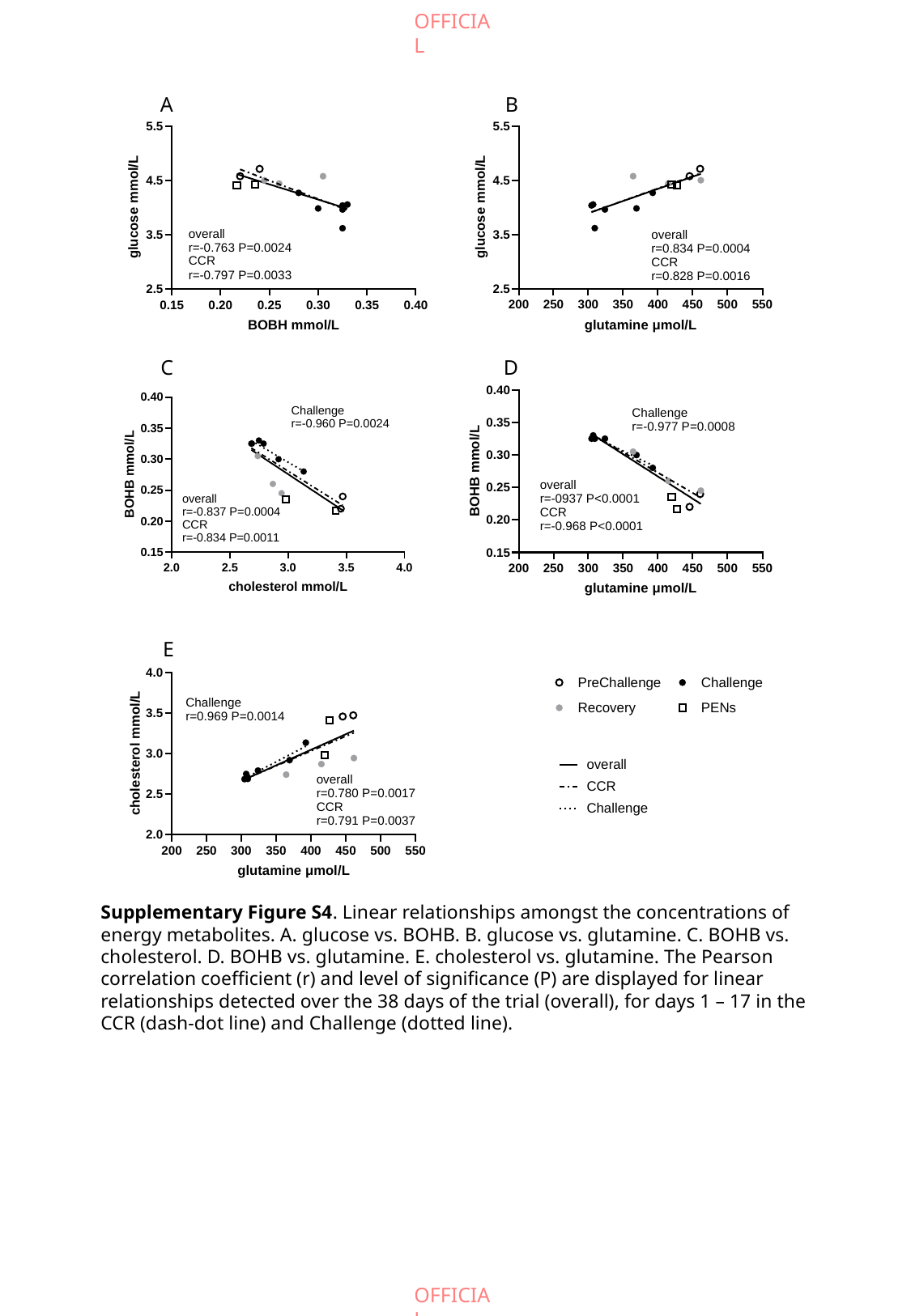

A
B
C
D
E
Supplementary Figure S4. Linear relationships amongst the concentrations of energy metabolites. A. glucose vs. BOHB. B. glucose vs. glutamine. C. BOHB vs. cholesterol. D. BOHB vs. glutamine. E. cholesterol vs. glutamine. The Pearson correlation coefficient (r) and level of significance (P) are displayed for linear relationships detected over the 38 days of the trial (overall), for days 1 – 17 in the CCR (dash-dot line) and Challenge (dotted line).

## Slide 5
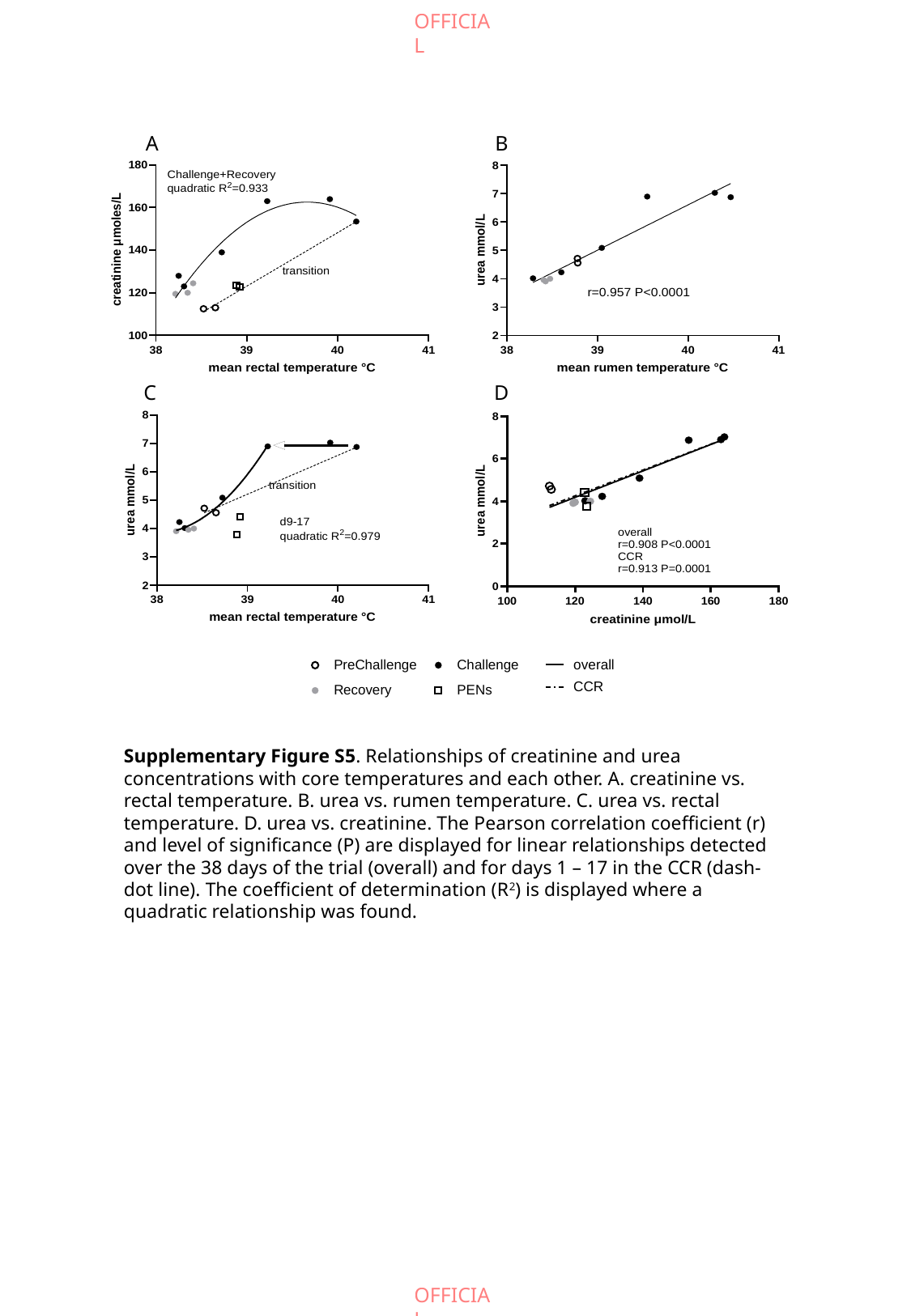

A
B
C
D
Supplementary Figure S5. Relationships of creatinine and urea concentrations with core temperatures and each other. A. creatinine vs. rectal temperature. B. urea vs. rumen temperature. C. urea vs. rectal temperature. D. urea vs. creatinine. The Pearson correlation coefficient (r) and level of significance (P) are displayed for linear relationships detected over the 38 days of the trial (overall) and for days 1 – 17 in the CCR (dash-dot line). The coefficient of determination (R2) is displayed where a quadratic relationship was found.

## Slide 6
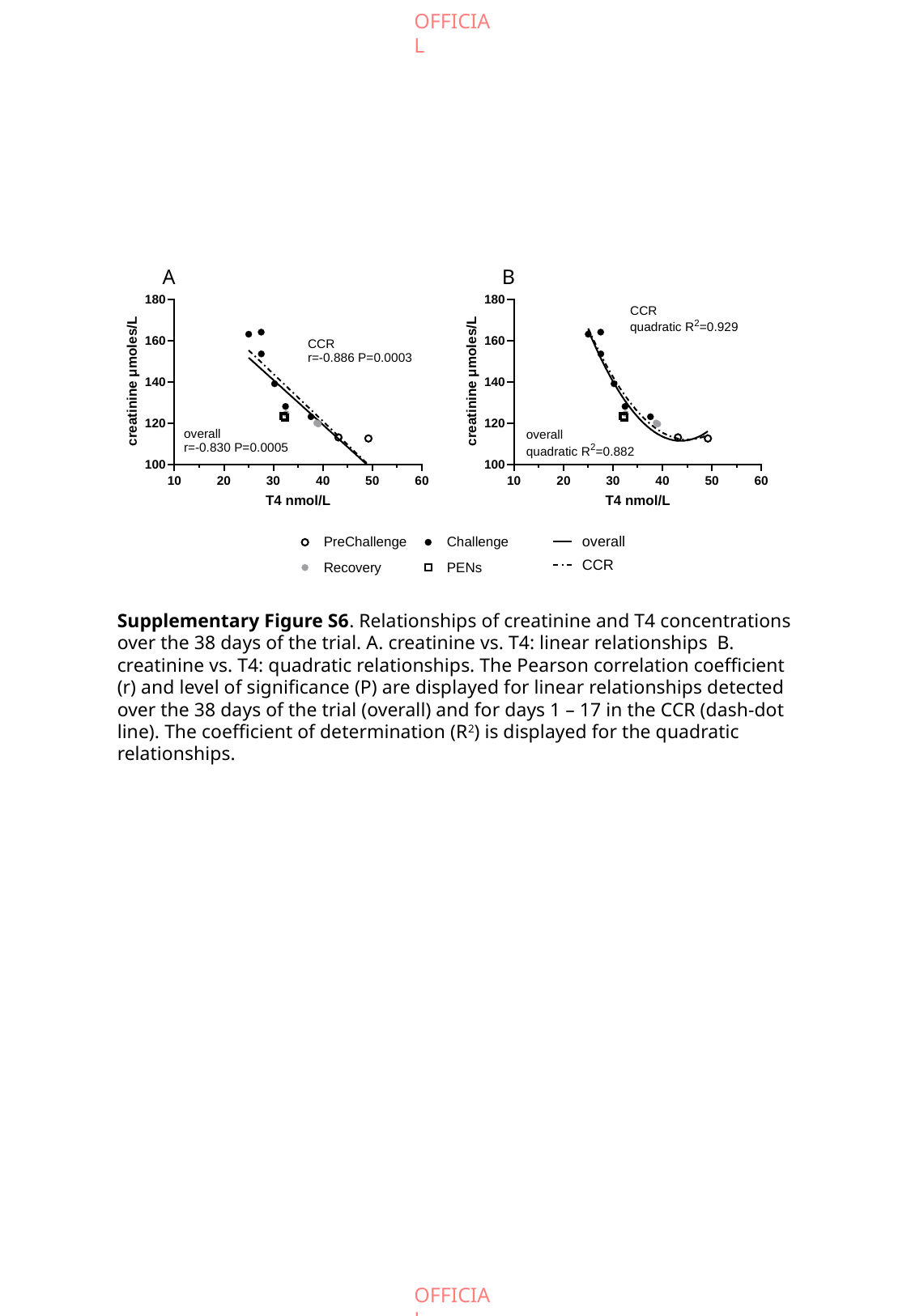

A
B
Supplementary Figure S6. Relationships of creatinine and T4 concentrations over the 38 days of the trial. A. creatinine vs. T4: linear relationships B. creatinine vs. T4: quadratic relationships. The Pearson correlation coefficient (r) and level of significance (P) are displayed for linear relationships detected over the 38 days of the trial (overall) and for days 1 – 17 in the CCR (dash-dot line). The coefficient of determination (R2) is displayed for the quadratic relationships.

## Slide 7
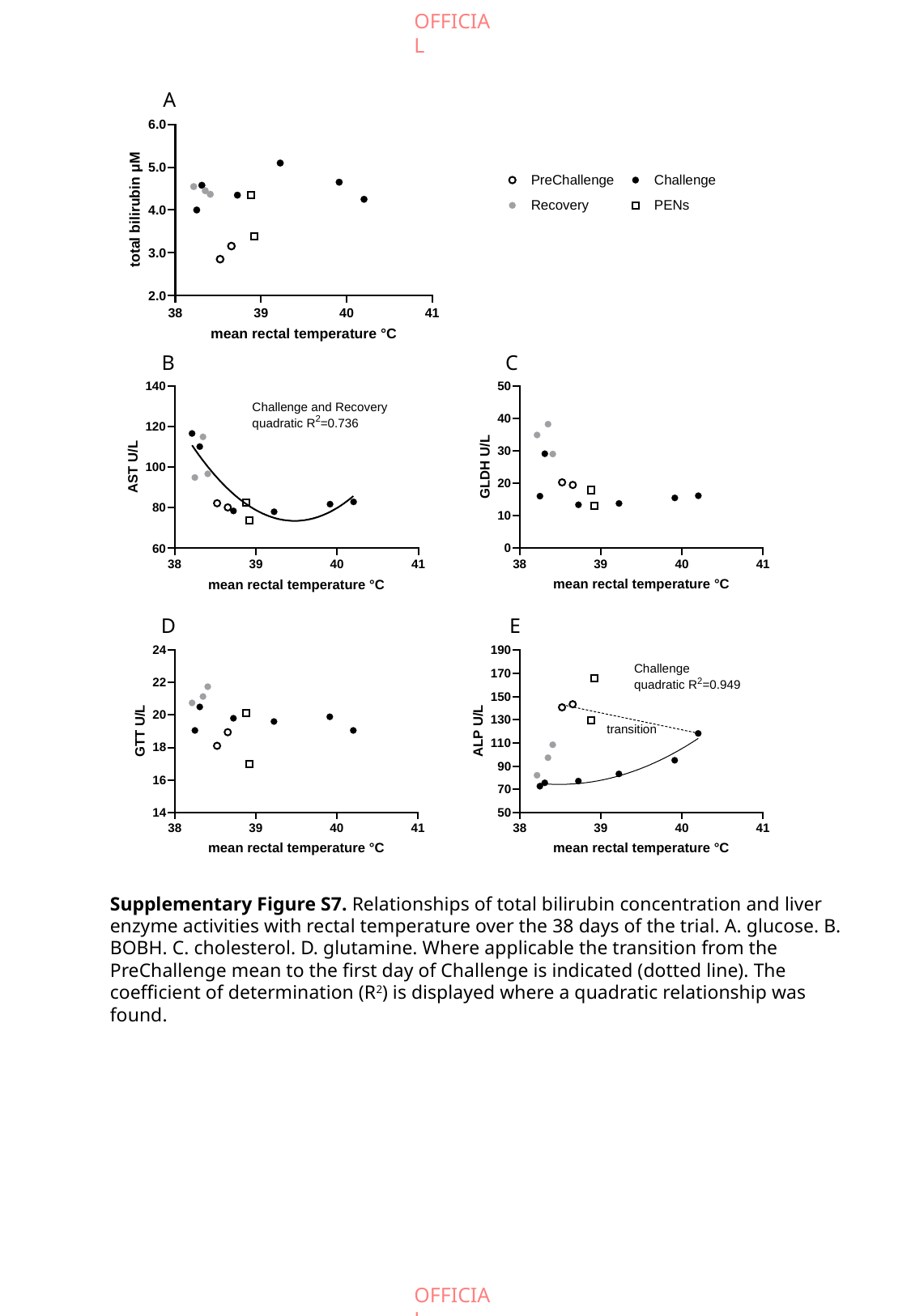

A
B
C
D
E
Supplementary Figure S7. Relationships of total bilirubin concentration and liver enzyme activities with rectal temperature over the 38 days of the trial. A. glucose. B. BOBH. C. cholesterol. D. glutamine. Where applicable the transition from the PreChallenge mean to the first day of Challenge is indicated (dotted line). The coefficient of determination (R2) is displayed where a quadratic relationship was found.

## Slide 8
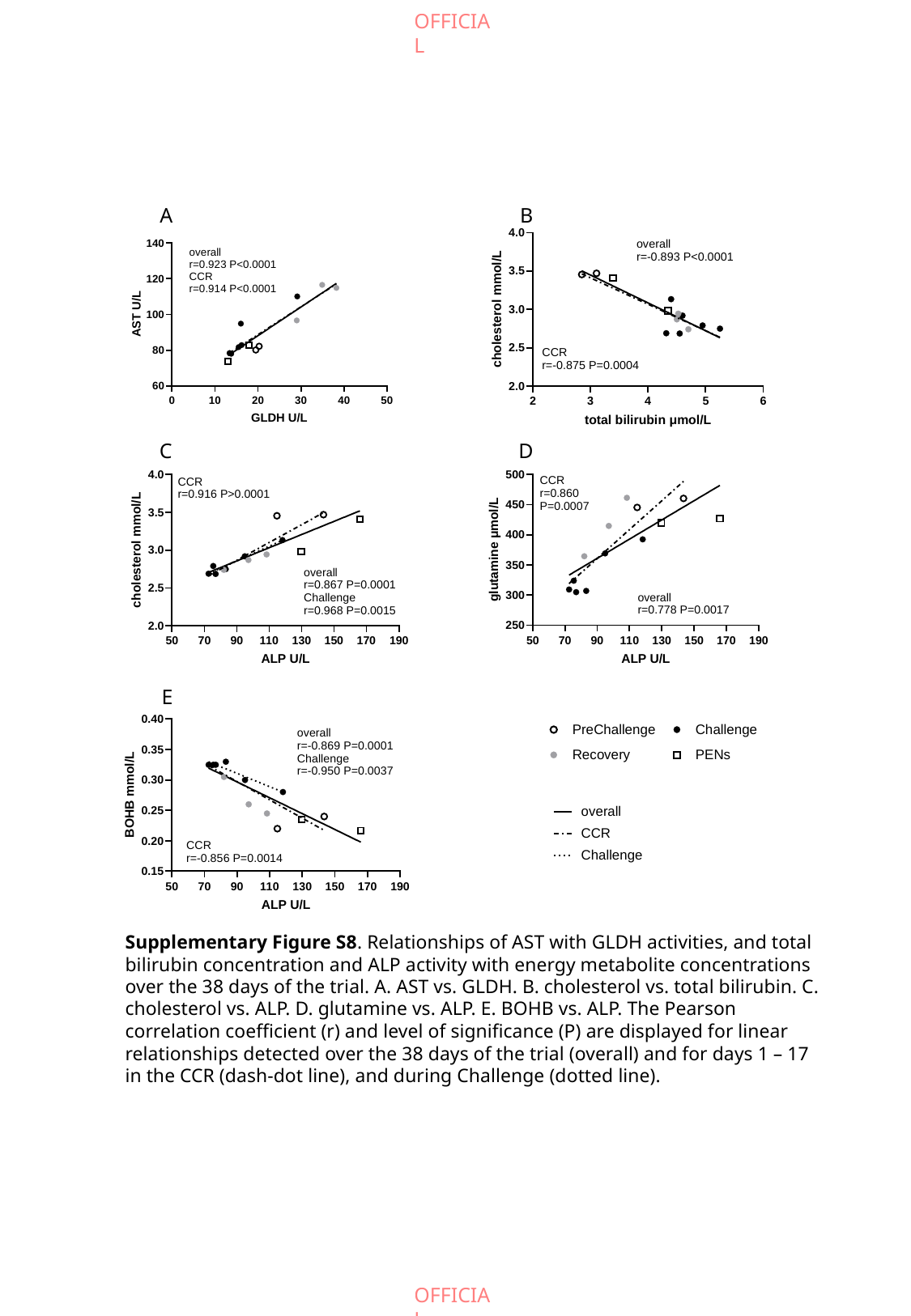

B
A
C
D
E
Supplementary Figure S8. Relationships of AST with GLDH activities, and total bilirubin concentration and ALP activity with energy metabolite concentrations over the 38 days of the trial. A. AST vs. GLDH. B. cholesterol vs. total bilirubin. C. cholesterol vs. ALP. D. glutamine vs. ALP. E. BOHB vs. ALP. The Pearson correlation coefficient (r) and level of significance (P) are displayed for linear relationships detected over the 38 days of the trial (overall) and for days 1 – 17 in the CCR (dash-dot line), and during Challenge (dotted line).

## Slide 9
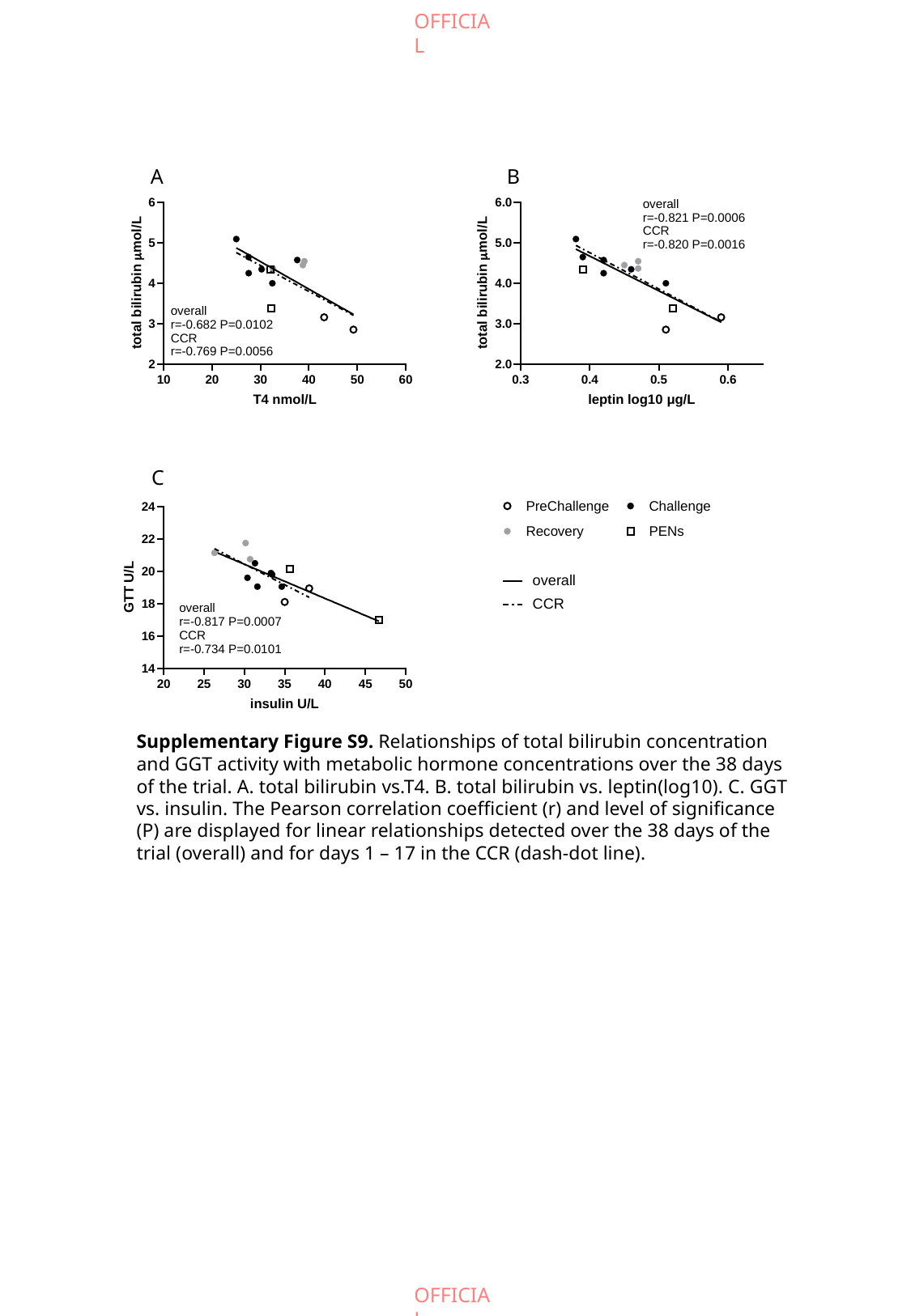

A
B
C
Supplementary Figure S9. Relationships of total bilirubin concentration and GGT activity with metabolic hormone concentrations over the 38 days of the trial. A. total bilirubin vs.T4. B. total bilirubin vs. leptin(log10). C. GGT vs. insulin. The Pearson correlation coefficient (r) and level of significance (P) are displayed for linear relationships detected over the 38 days of the trial (overall) and for days 1 – 17 in the CCR (dash-dot line).

## Slide 10
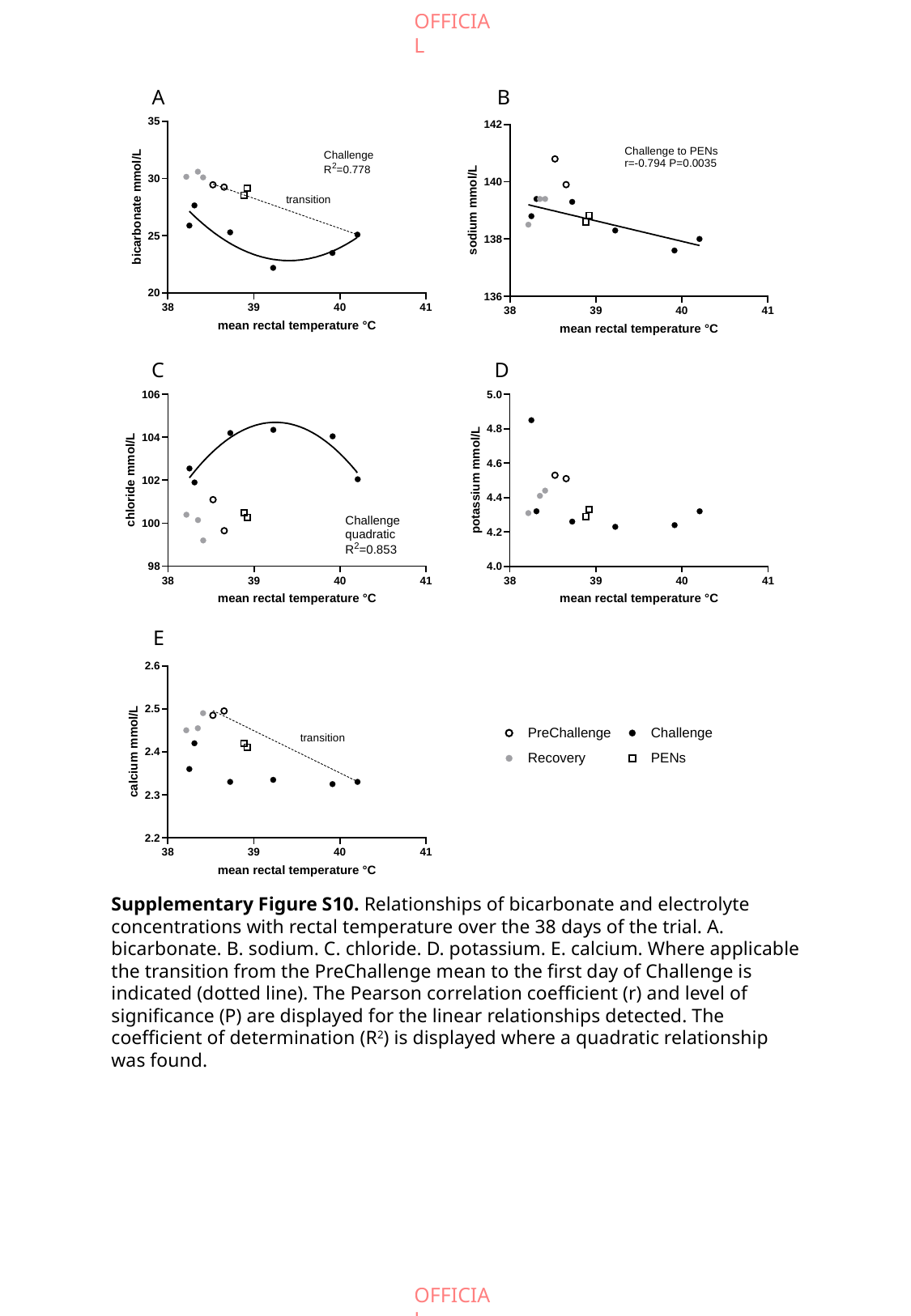

A
B
C
D
E
Supplementary Figure S10. Relationships of bicarbonate and electrolyte concentrations with rectal temperature over the 38 days of the trial. A. bicarbonate. B. sodium. C. chloride. D. potassium. E. calcium. Where applicable the transition from the PreChallenge mean to the first day of Challenge is indicated (dotted line). The Pearson correlation coefficient (r) and level of significance (P) are displayed for the linear relationships detected. The coefficient of determination (R2) is displayed where a quadratic relationship was found.

## Slide 11
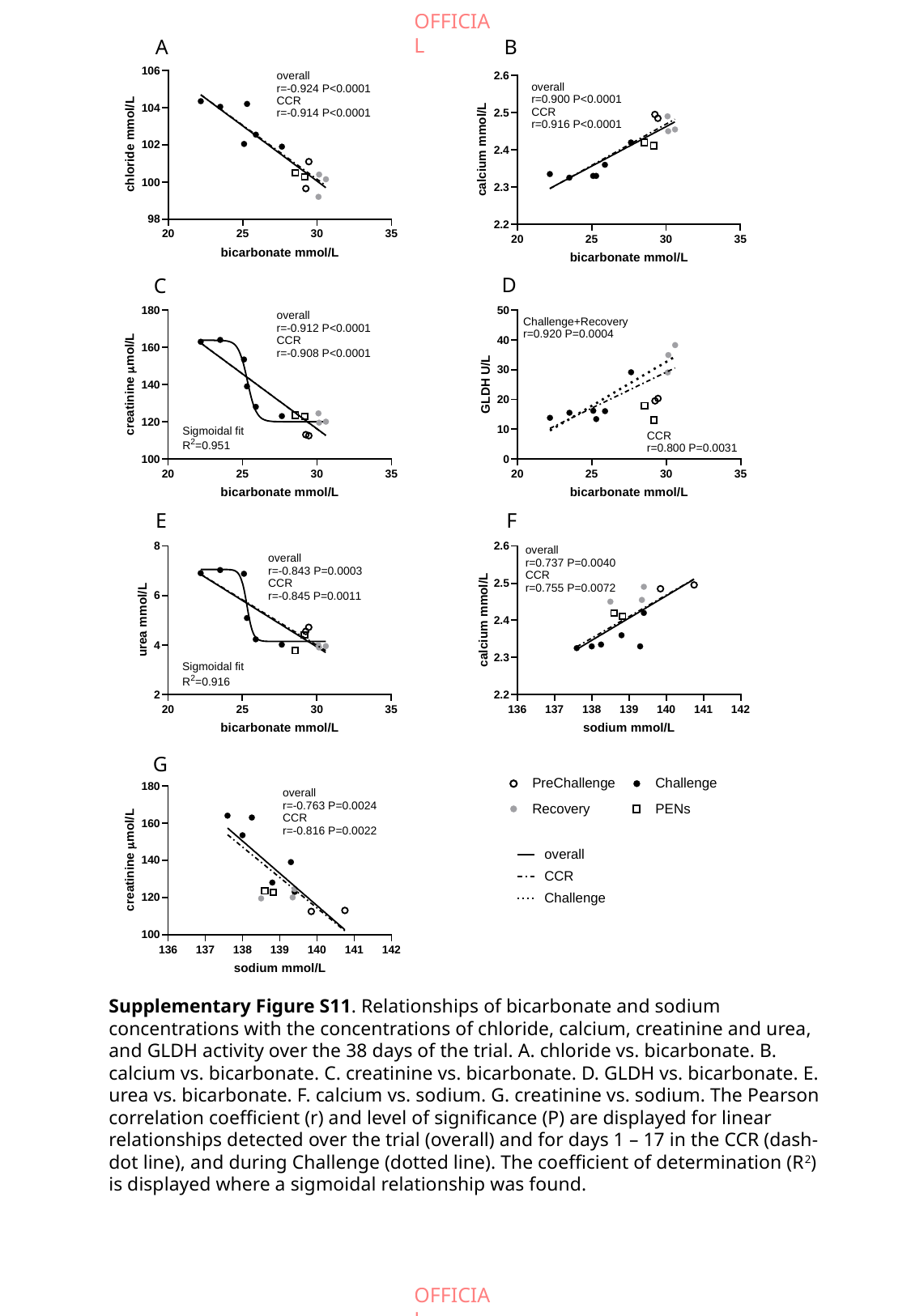

A
B
D
C
E
F
G
Supplementary Figure S11. Relationships of bicarbonate and sodium concentrations with the concentrations of chloride, calcium, creatinine and urea, and GLDH activity over the 38 days of the trial. A. chloride vs. bicarbonate. B. calcium vs. bicarbonate. C. creatinine vs. bicarbonate. D. GLDH vs. bicarbonate. E. urea vs. bicarbonate. F. calcium vs. sodium. G. creatinine vs. sodium. The Pearson correlation coefficient (r) and level of significance (P) are displayed for linear relationships detected over the trial (overall) and for days 1 – 17 in the CCR (dash-dot line), and during Challenge (dotted line). The coefficient of determination (R2) is displayed where a sigmoidal relationship was found.

## Slide 12
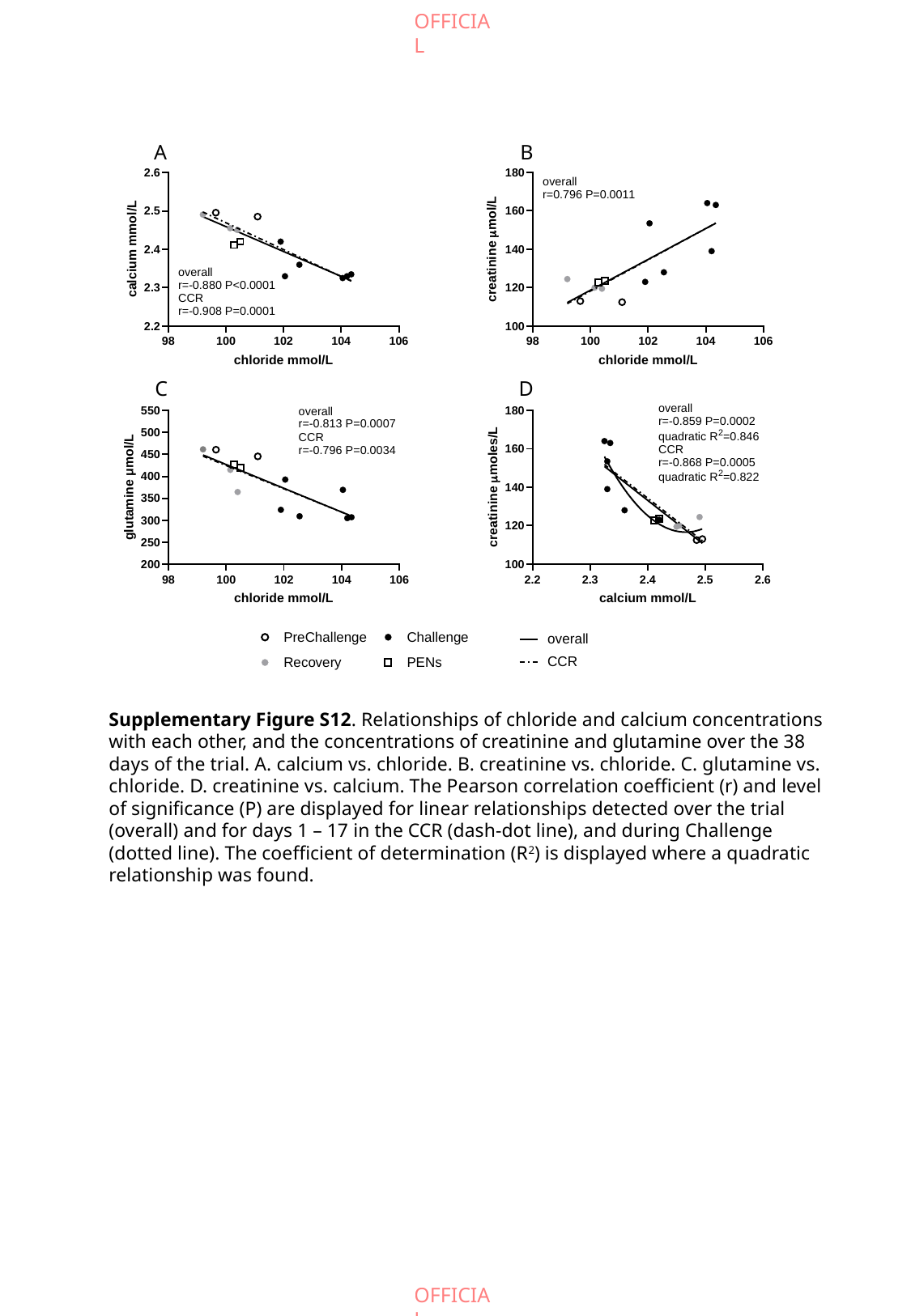

A
B
C
D
Supplementary Figure S12. Relationships of chloride and calcium concentrations with each other, and the concentrations of creatinine and glutamine over the 38 days of the trial. A. calcium vs. chloride. B. creatinine vs. chloride. C. glutamine vs. chloride. D. creatinine vs. calcium. The Pearson correlation coefficient (r) and level of significance (P) are displayed for linear relationships detected over the trial (overall) and for days 1 – 17 in the CCR (dash-dot line), and during Challenge (dotted line). The coefficient of determination (R2) is displayed where a quadratic relationship was found.

## Slide 13
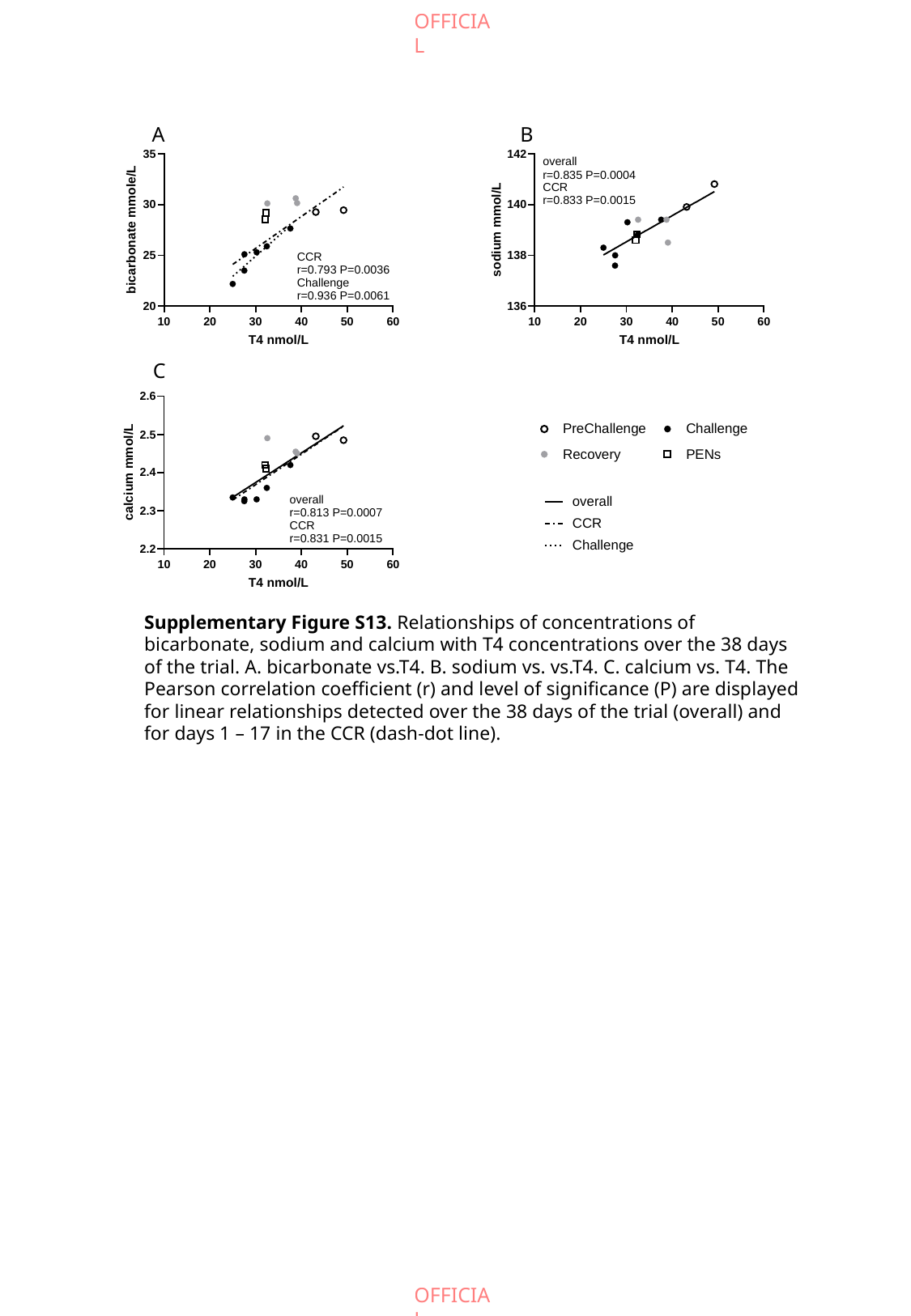

A
B
C
Supplementary Figure S13. Relationships of concentrations of bicarbonate, sodium and calcium with T4 concentrations over the 38 days of the trial. A. bicarbonate vs.T4. B. sodium vs. vs.T4. C. calcium vs. T4. The Pearson correlation coefficient (r) and level of significance (P) are displayed for linear relationships detected over the 38 days of the trial (overall) and for days 1 – 17 in the CCR (dash-dot line).

## Slide 14
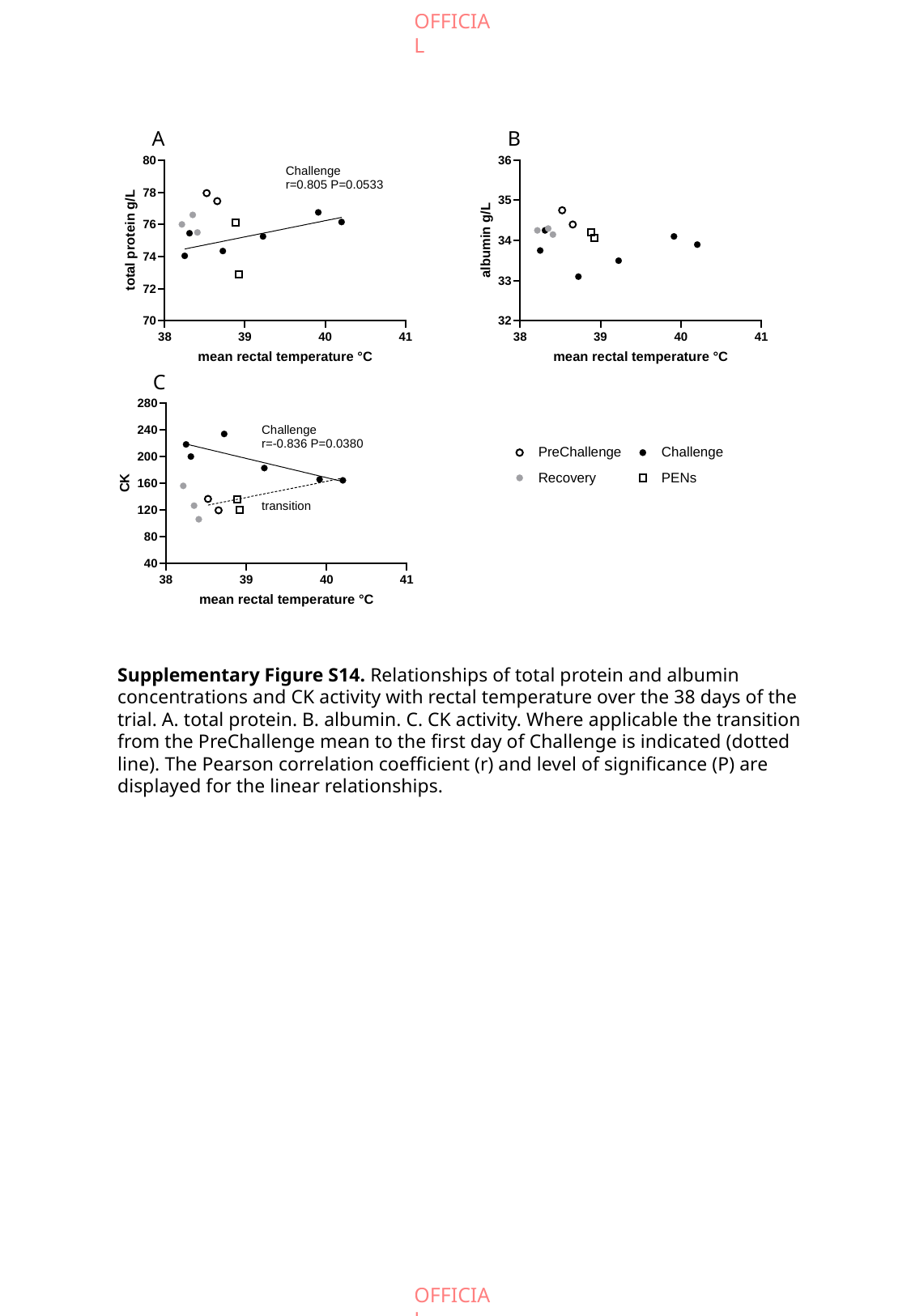

A
B
C
Supplementary Figure S14. Relationships of total protein and albumin concentrations and CK activity with rectal temperature over the 38 days of the trial. A. total protein. B. albumin. C. CK activity. Where applicable the transition from the PreChallenge mean to the first day of Challenge is indicated (dotted line). The Pearson correlation coefficient (r) and level of significance (P) are displayed for the linear relationships.

## Slide 15
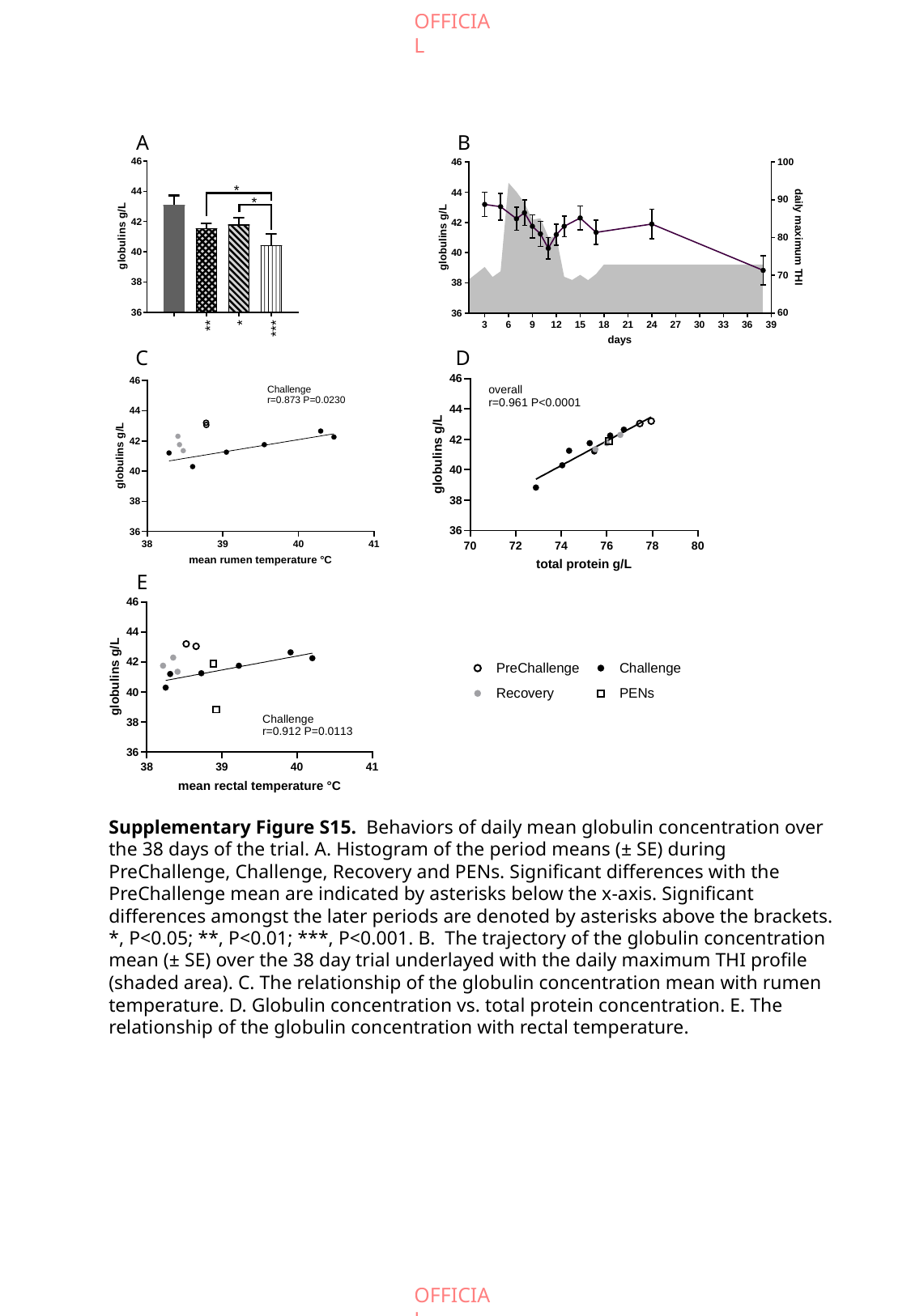

A
B
C
D
E
Supplementary Figure S15. Behaviors of daily mean globulin concentration over the 38 days of the trial. A. Histogram of the period means (± SE) during PreChallenge, Challenge, Recovery and PENs. Significant differences with the PreChallenge mean are indicated by asterisks below the x-axis. Significant differences amongst the later periods are denoted by asterisks above the brackets. *, P<0.05; **, P<0.01; ***, P<0.001. B. The trajectory of the globulin concentration mean (± SE) over the 38 day trial underlayed with the daily maximum THI profile (shaded area). C. The relationship of the globulin concentration mean with rumen temperature. D. Globulin concentration vs. total protein concentration. E. The relationship of the globulin concentration with rectal temperature.

## Slide 16
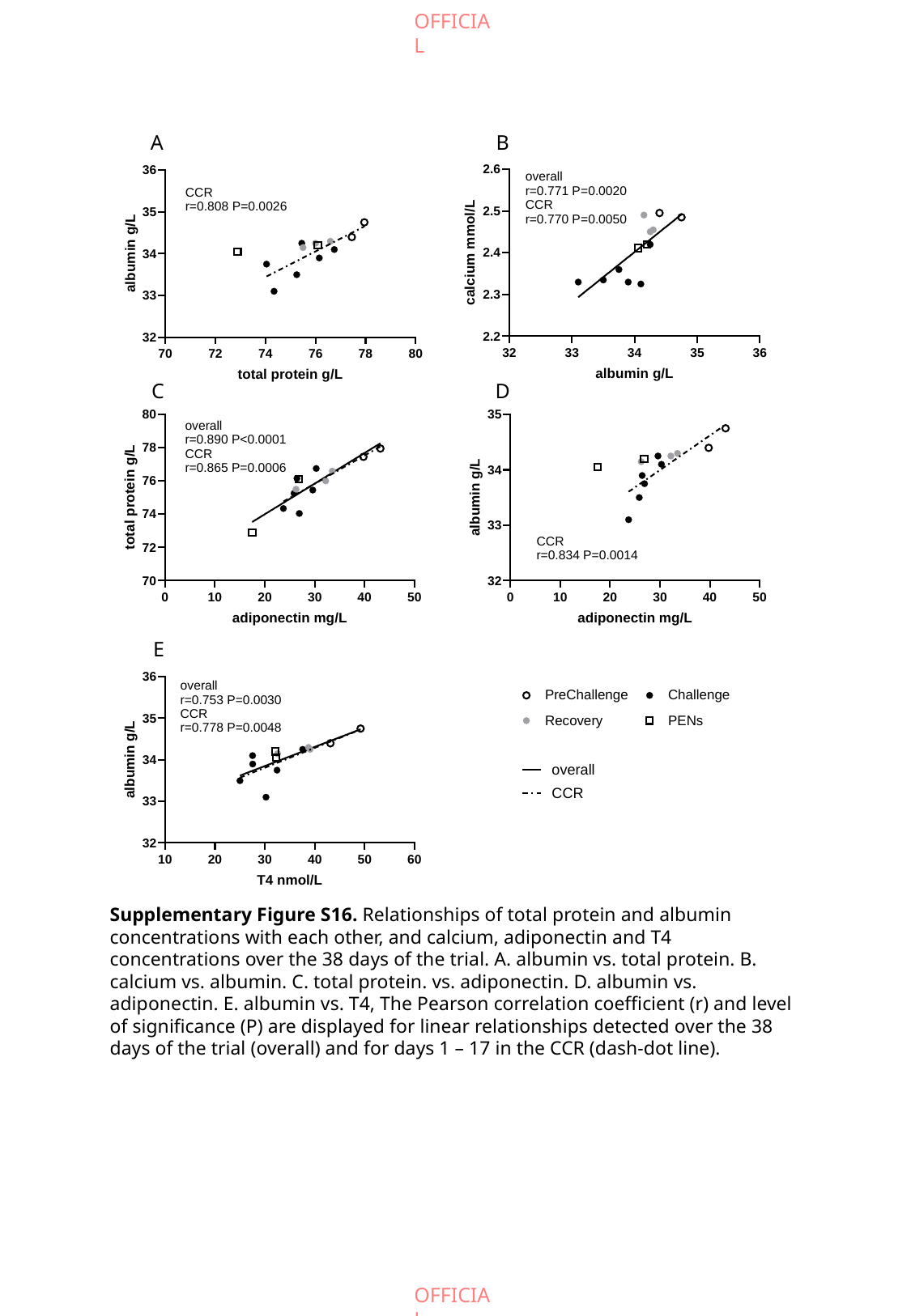

A
B
C
D
E
Supplementary Figure S16. Relationships of total protein and albumin concentrations with each other, and calcium, adiponectin and T4 concentrations over the 38 days of the trial. A. albumin vs. total protein. B. calcium vs. albumin. C. total protein. vs. adiponectin. D. albumin vs. adiponectin. E. albumin vs. T4, The Pearson correlation coefficient (r) and level of significance (P) are displayed for linear relationships detected over the 38 days of the trial (overall) and for days 1 – 17 in the CCR (dash-dot line).

## Slide 17
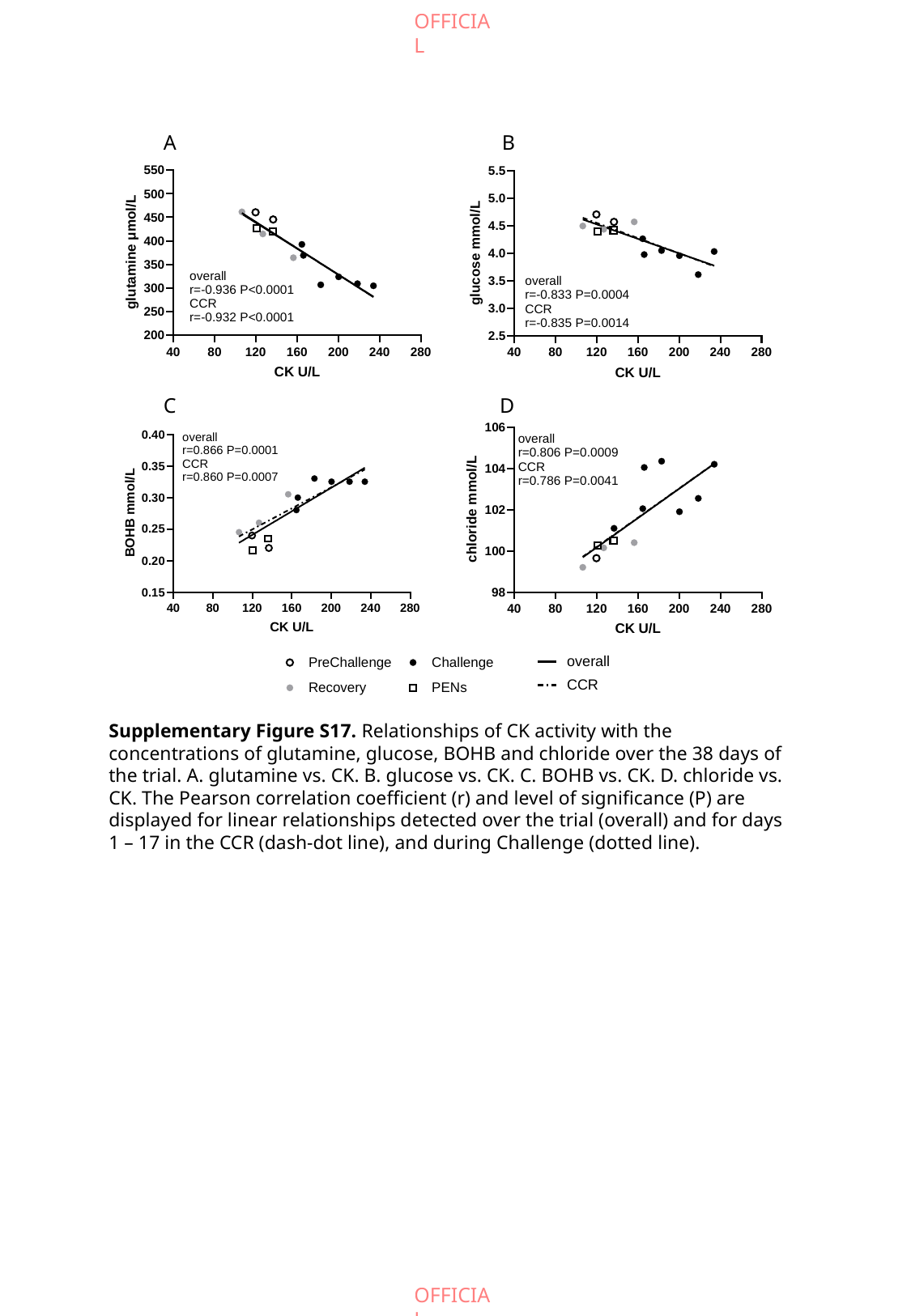

A
B
C
D
Supplementary Figure S17. Relationships of CK activity with the concentrations of glutamine, glucose, BOHB and chloride over the 38 days of the trial. A. glutamine vs. CK. B. glucose vs. CK. C. BOHB vs. CK. D. chloride vs. CK. The Pearson correlation coefficient (r) and level of significance (P) are displayed for linear relationships detected over the trial (overall) and for days 1 – 17 in the CCR (dash-dot line), and during Challenge (dotted line).

## Slide 18
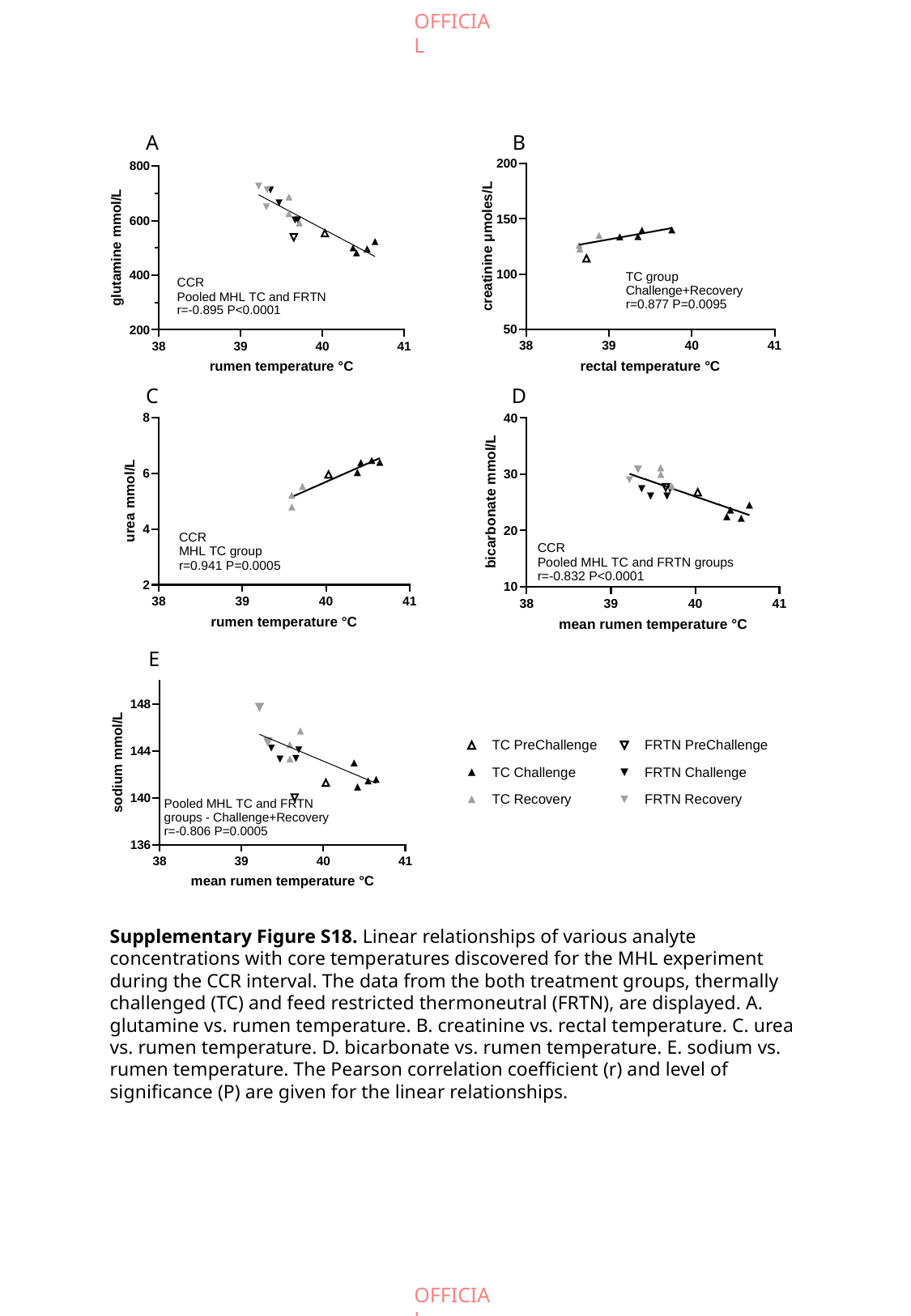

A
B
C
D
E
Supplementary Figure S18. Linear relationships of various analyte concentrations with core temperatures discovered for the MHL experiment during the CCR interval. The data from the both treatment groups, thermally challenged (TC) and feed restricted thermoneutral (FRTN), are displayed. A. glutamine vs. rumen temperature. B. creatinine vs. rectal temperature. C. urea vs. rumen temperature. D. bicarbonate vs. rumen temperature. E. sodium vs. rumen temperature. The Pearson correlation coefficient (r) and level of significance (P) are given for the linear relationships.
