# Supplementary material for: Stress and Strain: Differentiating the Responses to High and Moderate Heat Loads and Subsequent Recovery in Grain-Fed Feedlot Steers—Plasma Biochemistry
Source: Animals (Basel). 2026 Apr 30;16(9):1379. doi: 10.3390/ani16091379 (PMC13162586; doi:10.3390/ani16091379)
Supplement: Supplementary file 1 [file animals-16-01379-s001.zip › CC7+8 biochem Supplementary Tables.pdf]

Supplementary Table S1. The daily maximum and minimum air temperature, % relative humidity (%RH) and temperature humidity index (THI) for the 17 days in the climate controlled rooms. The values given are the daily means experienced by the two cohorts.

day	Maximum air temperature (°C)	Minimum air temperature (°C)	Maximum %RH	Minimum %RH	Maximum THI	Minimum THI
1	20.82	19.30	94.02	61.92	72.24	64.98
2	21.56	19.44	96.14	58.43	71.08	65.32
3	23.69	19.36	95.77	58.66	94.53	68.54
4	21.07	19.35	96.21	59.12	92.13	79.63
5	22.71	19.48	94.04	59.03	89.21	78.60
6	40.92	21.67	85.74	41.97	84.76	73.21
7	39.54	28.69	83.10	43.36	85.12	69.44
8	38.30	28.12	79.08	43.50	80.24	66.76
9	34.94	24.66	79.60	45.35	80.20	66.54
10	34.31	22.29	84.85	47.42	69.66	65.09
11	30.39	20.30	85.28	50.65	68.81	64.98
12	30.69	20.13	84.84	42.51	70.16	65.11
13	21.43	19.44	92.67	53.90	68.74	65.09
14	21.17	19.36	89.61	57.81	70.39	65.12
15	22.33	19.43	92.07	58.84	72.24	64.98
16	20.92	19.45	91.72	58.41	71.08	65.32
17	21.55	19.15	96.79	57.63	94.53	68.54

During the 20 day PENs period, the overall mean (± SD) maximum and minimum air temperatures were 24.5 ± 5.3 °C and 15 0 ± 2.1 °C respectively; the overall mean (± SD) maximum and minimum %RH were 90.5 ± 3.2% and 51.7 ± 7.0% respectively; and the overall mean (± SD) maximum and minimum THI were 72.7 ± 2.1 and 58.9 ± 3.5% respectively.

Supplementary Table S2. Linear relationships detected amongst the daily mean plasma concentrations of the analytes. The Pearson correlation coefficient (r) and level of significance (bracketed) are listed. The interval refers the days of the trial the relationships were assessed; overall, 38 days; CCR, the 17 days in the climate controlled rooms and inclusive of the PreChallenge, Challenge and Recovery periods; Challenge equates to the Challenge period.

analyte	interval	glucose	BOBH		cholesterol			
BOBH	overall	-0.763 (0.0024)	-		-			
	CCR	-0.797 (0.0033)	-		-			
cholesterol	overall	-	-0.837 (0.0004)		-			
	CCR	-	-0.843 (0.0011)		-			
	Challenge	-	-0.960 (0.0024)		-			
glutamine	overall	0.834 (0.0004)	-0.937 (<0.0001)		0.780 (0.0017)			
	CCR	0.828 (0.0016)	-0.968 (<0.0001)		0.791 (0.0037)			
	Challenge	-	-0.977 (0.0008)		0.969 (0.0014)			
**analyte**	**interval**	**GLDH**	**BOBH**		**cholesterol**	**glutamine**		
Total bilirubin	overall	-	-		-0.893 (<0.0001)	-		
	CCR	-	-		-0.875 (0.0004)	-		
AST	overall	0.923 (<0.0001)	-		-	-		

	CCR	0.914 (<0.0001)	-		-	-		
	Challenge	0.923 (0.0087)	-		-	-		
ALP	overall	-	-0.869 (0.0001)		0.867 (0.0001)	0.778 (0.0017)		
	CCR	-	-0.856 (0.0014)		0.916 (<0.0001)	0.860 (0.0007)		
	Challenge	-	-0.950 (0.0037)		0.968 (0.0015)	0.923 (0.0086)		
**analyte**	**interval**	**chloride**	**calcium**		**creatinine**	**GLDH**	**glutamine**	**urea**
bicarbonate	overall	-0.924 (<0.0001)	0.900 (<0.0001)		-0.912 (<0.0001)	-	-	-0.843 (0.0003)
	CCR	-0.921 (<0.0001)	0.916 (<0.0001)		-0.908 (<0.0001)	0.800 (0.0031)	-	-0.845 (0.0011)
	Challenge	-	-		-0.914 (0.0109)	0.920 (0.0004)	-	-0.837 (0.0377)
sodium	overall	-	0.737 (0.0040)		-0.763 (0.0024)	-	-	-
	CCR	-	0.755 (0.0072)		-0.816 (0.0022)	-	-	-
chloride	overall	-	-0.880 (<0.0001)		0.796 (0.0011)	-	-0.813 (0.0007)	-
	CCR	-	-0.908 (0.0001)		0.787 (0.0040)	-	-0.796 (0.0034)	-
calcium	overall	-	-		-0.859 (0.0002)	-	-	-
	CCR	-	-		-0.868 (0.0005)	-	-	-
**analyte**	**interval**	**albumin**	**calcium**					
	overall	0.637 (0.0193)	-					

Total protein	CCR	0.808 (0.0026)	-					
	Challenge	-	-					
albumin	overall	-	0.771 (0.0022)					
	CCR	-	0.770 (0.0050)					
**analyte**	**interval**	**glutamine**	**glucose**		**BOBH**	**chloride**		
CK	overall	-0.936 (<0.0001)	-0.833 (0.0004)		0.866 (0.0001)	0.806 (0.0009)		
	CCR	-0.932 (<0.0001)	-0.835 (0.0014)		0.860 (0.0007)	0.786 (0.00041)		

Supplementary Table S3. Visualisation of the notable correlations amongst analytes as presented in Supplementary Table S2.

Overall

	glucose	glutamine	BOBH	cholesterol
glucose		0.834	-0.763	0.636
glutamine	0.834		-0.937	0.780
BOBH	-0.763	-0.937		-0.834
cholesterol	0.636	0.780	-0.834	

CCR

	glucose	glutamine	BOBH	cholesterol
glucose		0.828	-0.797	0.655
glutamine	0.828		-0.968	0.791
BOBH	-0.797	-0.968		-0.843
cholesterol	0.655	0.791	-0.843	

Challenge

	glucose	glutamine	BOBH	cholesterol
glucose		0.592	-0.517	0.286
glutamine	0.592		-0.970	0.835
BOBH	-0.517	-0.970		-0.883
cholesterol	0.286	0.835	-0.883	

Overall

	GLDH	B0BH	cholesterol	glutamine
Total bilirubin	0.207	0.764	-0.893	-0.671
AST	0.923	0.258	-0.473	-0.104
ALP	-0.259	-0.869	0.867	0.778

CCR

	GLDH	B0BH	cholesterol	glutamine
bili_T	0.097	0.863	-0.878	-0.792
AST	0.924	0.115	-0.389	-0.043
ALP	-0.092	-0.840	0.930	0.891

Challenge

	GLDH	B0BH	cholesterol	glutamine
bili_T	0.247	0.490	-0.257	-0.370
AST	0.923	0.252	-0.163	-0.153
ALP	-0.225	-0.950	0.968	0.923

Overall

	chloride	calcium	creatinine	GLDH	glutamine	urea
bicarbonate	-0.924	0.900	-0.912	0.664	0.686	-0.843
sodium	-0.524	0.737	-0.763	0.252	0.431	-0.575
chloride		-0.880	0.796	-0.554	-0.813	0.711
calcium	-0.880		-0.859	0.609	0.739	-0.717

CCR

	chloride	calcium	creatinine	GLDH	glutamine	urea
bicarbonate	-0.924	0.900	-0.912	0.664	0.686	-0.843
sodium	-0.524	0.737	-0.763	0.252	0.431	-0.575
chloride		-0.880	0.796	-0.554	-0.813	0.711
calcium	-0.880		-0.859	0.609	0.739	-0.717

Challenge

	chloride	calcium	creatinine	GLDH	glutamine	urea
bicarbonate	-0.773	0.769	-0.914	0.742	-0.081	-0.837
sodium	-0.251	0.651	-0.864	0.476	-0.695	-0.882
chloride		-0.641	0.603	-0.679	-0.323	0.459
calcium	-0.641		-0.776	0.943	-0.296	-0.758

Overall

	albumin	calcium
T_protein	0.637	0.415
albumin		0.771

Challenge

	albumin	calcium
T_protein	0.808	0.535
albumin		0.770

Overall

	glutamine	glucose	BOBH	chloride
CK	-0.936	-0.833	0.866	0.806

CCR

	glutamine	glucose	BOBH	chloride
CK	-0.932	-0.835	0.859	0.786

Supplementary Table S4. Linear relationships detected between the daily mean plasma concentrations of the analytes and the daily mean concentrations of the metabolic hormones previously reported upon [6]. The Pearson correlation coefficient (r) and level of significance (bracketed) are listed. The interval refers the days of the trial the relationships were assessed; overall, 38 days; CCR, the 17 days in the climate controlled rooms and inclusive of the PreChallenge, Challenge and Recovery periods; Challenge equates to the Challenge period.

**analyte**	**interval**	**T3**	**T3(log10)**	
BOBH	overall	-0.777 (0.0018)	-0.796 (0.0011)	-
	CCR	-0.702 (0.0160)	-0.710 (0.0142)	-
		**T4**	**leptin(log10)**	**insulin**
creatinine	overall	-0.830 (0.0005)	-	-
	CCR	-0.886 (0.0003)	-	-
	Challenge	-0.925 (0.0082)	-	-
total bilirubin	overall	-0.682 (0.0102)	-0.821 (0.0006)	-
	CCR	-0.769 (0.0056)	-0.820 (0.0016)	-
	Challenge	-	-0.860 (0.0279)	-
GGT	overall	-	-	-0.817 (0.0007)
	CCR	-	-	-0.734 (0.0101)
**analyte**	**interval**	**T4**	**adiponectin**	
bicarbonate	overall	0.730 (0.0047)	-	
	CCR	0.793 (0.0036)	-	
	Challenge	0.936 (0.0061)	-	
sodium	overall	0.835 (0.0004)	-	
	CCR	0.833 (0.0015)	-	
calcium	overall	0.813 (0.0007)	-	
	CCR	0.831 (0.0015)	-	
	Challenge	-	0.890 (<0.0001)	
total protein	overall	-	0.865 (0.0006	
albumin	overall	0.753 (0.0030)	0.834 (0.0014	
	CCR	0.778 (0.0048)	-	

Supplementary Table S5. Visualisation of the notable correlations between analytes and hormone concentrations as presented in Supplementary Table S4.

Overall

	T3	T3 log 10
BOBH	-0.776	-0.796

CCR

	T3	T3 log 10
BOBH	-0.702	-0.711

Overall

	T4	leptin (log10)	insulin
creatinine	-0.829	-0.676	-0.223
Total bilirubin	-0.682	-0.821	-0.641
GTT	-0.102	-0.424	-0.817

CCR

	T4	leptin (log10)	insulin
creatinine	-0.886	-0.770	-0.148
Total bilirubin	-0.769	-0.829	-0.686
GTT	-0.201	-0.316	-0.734

Challenge

	T4	leptin (log10)	insulin
creatinine	-0.925	-0.719	-0.327
Total bilirubin	-0.398	-0.860	-0.737
GTT	0.494	-0.402	-0.297

Overall

	T4	adiponectin
bicarbonate	0.730	0.328
sodium	0.835	0.576
calcium	0.813	0.556
total protein	0.536	0.890
albumin	0.753	0.672

CCR

	T4	adiponectin
bicarbonate	0.730	0.328
sodium	0.835	0.576
calcium	0.813	0.556
total protein	0.536	0.890
albumin	0.753	0.672

Challenge

	T4	adiponectin
bicarbonate	0.936	0.176
sodium	0.749	-0.319
calcium	0.901	0.433
total protein	-0.326	0.630
albumin	0.391	0.932
