# Supplementary material for: Stress and Strain: Differentiating the Responses to High and Moderate Heat Loads and Subsequent Recovery in Grain-Fed Feedlot Steers—Plasma Biochemistry
Source: Animals (Basel). 2026 Apr 30;16(9):1379. doi: 10.3390/ani16091379 (PMC13162586; doi:10.3390/ani16091379)
Supplement: Supplementary file 1 [file animals-16-01379-s001.zip › Supplementary Text.pdf]

Supplementary Text S1. PreChallenge Baseline Comparisons of the MHL and HHL experiments.

Figure 8 was produced to enable comparison of the MHL and HHL experiments at the level of the period mean concentrations. The PreChallenge data was used as reference in calculating the %change across the period means for both experiments. The MHL and HHL PreChallenge analyte concentrations or activities were assessed to understand if the steers in PreChallenge were in a similar baseline metabolic state. The PreChallenge analyte data of the MHL and HHL steers were compared by Welch's t-test to ascertain baseline similarity for each analyte. For the MHL experiment, the PreChallenge data was obtained from a single bleed performed on day 3 in the CCR (n=24, [3]). As outlined in Figure 2, there were two blood samplings conducted in PreChallenge for the HHL experiment, days 3 and 5 (n= 2 x 20). Nine of the 18 analyte concentrations did not differ between the experiments during PreChallenge (see table below).

Supplementary Text Table S1. Comparison of the analyte concentrations/activities of the MHL and HHL steers during PreChallenge.

	Mean ± SEM		Welsh's t-test	eta squared
	MHL	HHL	P value	R^2
glucose mmol/L	4.43 ± 0.105	4.64 ± 0.096	0.1459	0.0381
cholesterol mmol/L	2.68 ± 0.138	3.46 ± 0.142	0.0002	0.2123
glutamine µmol/L	546.7 ± 21.25	453.0 ± 14.66	0.0007	0.2293
creatinine µmol/L	114.1 ± 2.00	113.0 ± 1.40	0.6604	0.0044
urea mmol/	5.98 ± 0.132	4.64 ± 0.097	<0.0001	0.5943
total bilirubin µmol/L	2.83 ± 0.099	3.00 ± 0.122	0.2804	0.0191
AST U/L	72.94 ± 3.316	81.18 ± 1.826	0.0360	0.1133
GLDH U/L	12.08 ± 1.195	19.90 ± 1.828	0.0007	0.1744
GGT U/L	18.20 ± 0.581	18.53 ± 0.601	0.6953	0.0026
ALP U/L	118.4 ± 8.74	142.1 ± 8.80	0.0614	0.0590
bicarbonate mmol/L	27.23 ± 0.453	29.53 ± 0.339	0.0005	0.2300
sodium mmol/L	140.3 ± 1.19	140.7 ± 0.27	0.7691	0.0034
chloride mmol/L	101.2 ± 0.71	100.4 ± 0.29	0.2879	0.0244
potassium mmol/L	4.23 ± 0.061	4.52 ± 0.043	0.0002	0.2615
calcium mmol/L	2.34 ± 0.029	2.49 ± 0.010	<0.0001	0.4643
total protein g/L	74.5 ± 1.30	77.7 ± 0.61	0.0338	0.1277
albumin g/L	34.0 ± 0.45	34.6 ± 0.23	0.2247	0.0418
CK U/L	129.5 ± 10.6	128.4 ± 11.0	0.9398	0.0001

Glucose, creatinine, total bilirubin, sodium, chloride, and albumin concentrations and the activities of GGT and CK were not significantly different. The ALP activity tended toward significant difference (0.05 < P <0.1). The PreChallenge concentrations of the HHL steers were greater than those of the MHL steers for cholesterol, bicarbonate, potassium, calcium and total

protein concentrations. The GLDH and AST activities was also greater in the HHL steers, whereas the glutamine and urea concentrations were lower in the HHL steers in during PreChallenge. All values from all animals in both experiments were well within reference ranges for cattle [Queensland Government, 2019].

Despite the differences in some of the analyte means, most of the η^2 (eta squared) values were <0.25 (Supplementary Text Table S1). The η^2 values were >0.25 for urea, potassium and calcium indicating a great influence of group on these variables. The differences may reflect some physiological differences between the steers used in the two experiments. The body weights of the HHL steers at entry of the CCR was greater than the entry body weights of the MHL steers (603.4 ± 38.6 kg/head vs. 523.3 ± 22.9 kg/head respectively). This may explain the higher concentration of cholesterol in the HHL steers in PreChallenge due to their greater fat depots . their larger organ size, muscle mass and possibly bone mass may have contributed to the higher ALP, GLDH and AST activities. The finisher rations offered to the steers in the two experiments has the same Metabolizable Energy (ME; 13 MJ/kg) and %crude protein (15%). Percent fat differed; MHL finisher diet was at 1.6% whereas that of the HHL finisher diet was 5%. The increased fat content would have been due to the 9% cotton seed in the ration. The MHL steers had a greater mean DMI than the HHL steers during PreChallenge (11.09 ± 1.38 kg/day/head vs. 10.4 ± 1.99 kg/day/head respectively).

Queensland Government. Veterinary laboratory users guide. 6th ed.; Queensland Government, Brisbane, Australia, 2019; pp.137

Supplementary Text S2. Summary of findings described by Anderson et al., 2018 ([63])

Plasma bone ALP (BALP), osteocalcin (OCN) and CTX-1 are markers of bone turnover (Schini et al., 2023). The plasma levels of these markers were measured on day 5 (PreChallenge), days 7, 9 and 11 (Challenge) and days 13 and 15 (Recovery). During Challenge mean BALP activity fell 31-48% below the day 5 levels, and remained low on days 13 (48%) and 15 (30%). Mean OCN concentration decreased 15-20% over days 7 and 9 relative to day 5 concentration. The OCN concentration returned to PreChallenge concentration by day 11 and remained so over days 13 and 15. CTX-1 concentrations rose 38 and 77% relative to the day 5 concentration on days 7 and 9 respectively. The CTX-1 concentrations on days 11, 13 and 15 and were not different to the day 5 concentration.

Based on the results above, there was evidence for bone mobilisation over the first four days of Challenge. However, low plasma BALP activity persisted with return to thermoneutral conditions in Recovery mirroring the trajectory of plasma total ALP activity. This is suggestive of continuing defective bone mineralisation. The conclusion was that the high heat load reduced bone formation and triggered bone resorption in these rapidly growing young animals.

CTX-1: Carboxy-telopeptides of type I collagen

Anderson, S.; Steiger, N.; Sullivan, M.; Stockwell, S.; McCulloch, R.; Briscoe, S.; Cawdell-Smith, J.; Olm, J.; Gaughan, J.; Wijffels, G. Bone metabolism in growing cattle under severe heat stress. International Symposium on the Nutrition of Herbivores, Clermont-Ferrand, France, 2-6 September 2018.

Schini, M.; Vilaca, T.; Gossiel, F.; Salam, S.; Eastell, R. Bone turnover markers: basic biology to clinical applications. *Endocrine Reviews* **2023,** *44***,** 417-473**.** doi:10.1210/endrev/bnac031
